# The targetable nanoparticle BAF312@cRGD-CaP-NP represses tumor growth and angiogenesis by downregulating the S1PR1/P-STAT3/VEGFA axis in triple-negative breast cancer

**DOI:** 10.1186/s12951-021-00904-6

**Published:** 2021-05-31

**Authors:** Ke Gong, Juyang Jiao, Chaoqun Xu, Yang Dong, Dongxiao Li, Di He, De Zhao, Jian Yu, Ying Sun, Wei Zhang, Min Bai, Yourong Duan

**Affiliations:** 1 State Key Laboratory of Oncogenes and Related Genes, Shanghai Cancer Institute, Renji Hospital, School of Medicine, Shanghai Jiao Tong University, Shanghai, 200032 China; 2 Department of Bone and Joint Surgery, Department of Orthopedics, Renji Hospital, School of Medicine, Shanghai Jiao Tong University, Shanghai, 200011 China; 3 Sichuan Academy of Chinese Medicine Science, Chengdu, 610041 Sichuan China; 4 Department of Ultrasound, Shanghai General Hospital, Shanghai Jiao Tong University School of Medicine, 100 Haining Road, Shanghai, 200080 China

**Keywords:** Targeted nanoparticle, S1PR1, P-STAT3, VEGFA, BAF312, Triple-negative breast cancer

## Abstract

**Background:**

Overexpressed vascular endothelial growth factor A (VEGFA) and phosphorylated signal transducer and activator of transcription 3 (P-STAT3) cause unrestricted tumor growth and angiogenesis of breast cancer (BRCA), especially triple-negative breast cancer (TNBC). Hence, novel treatment strategy is urgently needed.

**Results:**

We found sphingosine 1 phosphate receptor 1 (S1PR1) can regulate P-STAT3/VEGFA. Database showed S1PR1 is highly expressed in BRCA and causes the poor prognosis of patients. Interrupting the expression of S1PR1 could inhibit the growth of human breast cancer cells (MCF-7 and MDA-MB-231) and suppress the angiogenesis of human umbilical vein endothelial cells (HUVECs) via affecting S1PR1/P-STAT3/VEGFA axis. Siponimod (BAF312) is a selective antagonist of S1PR1, which inhibits tumor growth and angiogenesis in vitro by downregulating the S1PR1/P-STAT3/VEGFA axis. We prepared pH-sensitive and tumor-targeted shell-core structure nanoparticles, in which hydrophilic PEG2000 modified with the cyclic Arg-Gly-Asp (cRGD) formed the shell, hydrophobic DSPE formed the core, and CaP (calcium and phosphate ions) was adsorbed onto the shell; the nanoparticles were used to deliver BAF312 (BAF312@cRGD-CaP-NPs). The size and potential of the nanoparticles were 109.9 ± 1.002 nm and − 10.6 ± 0.056 mV. The incorporation efficacy for BAF312 was 81.4%. Results confirmed BAF312@cRGD-CaP-NP could dramatically inhibit tumor growth and angiogenesis in vitro and in MDA-MB-231 tumor-bearing mice via downregulating the S1PR1/P-STAT3/VEGFA axis.

**Conclusions:**

Our data suggest a potent role for BAF312@cRGD-CaP-NPs in treating BRCA, especially TNBC by downregulating the S1PR1/P-STAT3/VEGFA axis.

**Graphic abstract:**

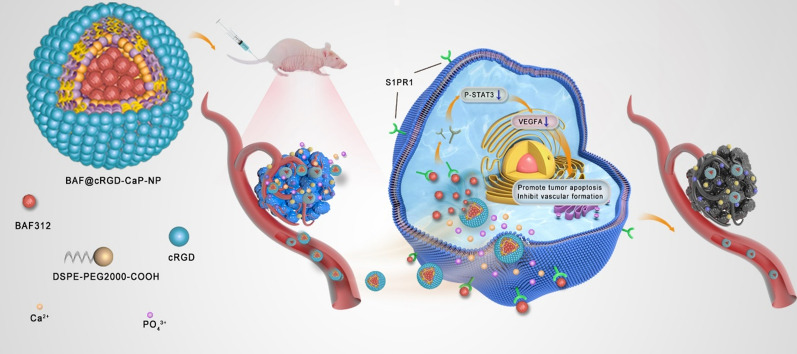

**Supplementary Information:**

The online version contains supplementary material available at 10.1186/s12951-021-00904-6.

## Background

Patients with triple-negative breast cancer (TNBC) show unsatisfactory outcomes, as TNBC is the most aggressive type of breast cancer (BRCA) [1]. Researches have gradually increased the understanding of the underlying mechanisms of breast cancer and have led to the development of effective strategies [2]. However, chemoresistance and tumor recurrence have hindered the recovery of patients with BRCA, especially those with TNBC [3, 4]. Therefore, it is urgently needed to identify novel treatment targets for patients with BRCA or TNBC.

Research has found that sphingosine 1 phosphate receptor 1 (S1PR1) plays a major role in tumor proliferation, angiogenesis and metastasis in breast cancer [5–7]. Moreover, studies have shown that S1PR1 induces chemoresistance in breast cancer [8, 9]. S1PR1 expression has been reported to be correlated with the progression of invasive breast carcinoma [10]. Besides, high expression of S1PR1 was correlated with a shorter time to tumor recurrence in BRCA patients [11]. These results indicate that S1PR1 could be a therapeutic target for patients with BRCA. Overexpressed vascular endothelial growth factor A (VEGFA) and highly activated signal transducer and activator of transcription 3 (STAT3) contribute to the unrestricted tumor growth and vascularization of BRCA, especially TNBC [12–15]. VEGFA is the most potent mediator of angiogenesis and promotes unrestricted tumor growth in TNBC [16, 17]. Angiogenesis determines the growth rate and progression of tumors by providing nutrition and niches for tumor cells to invade and further metastasize [18]. Research confirmed that a tumor can grow to no more than 2 mm^3^ in size without angiogenesis [19]. However, clinical trials of drugs that target the VEGFA signaling pathway has been disappointing. The antiangiogenic drug bevacizumab, a monoclonal antibody targeting VEGFA, has been proven to be less effective in treating breast cancer as a single agent [20, 21]. In addition, most patients gradually become insensitive to anti-VEGFA drugs for several reasons [21–23]. Thus, it is urgently needed to find a replacement strategy for overcoming chemoresistance in patients with TNBC. Earlier research indicated that the STAT3 protein binds to the VEGFA promoter in vivo and found that constitutive STAT3 activity upregulates VEGFA expression and tumor angiogenesis [24]. Moreover, a study found that inhibition of aberrant STAT3 activity suppresses VEGFA expression, thereby inducing the expression of the pro-apoptotic protein Bax [25]. Chang RX et al. analyzed the transcriptome characteristics of patients with TNBC and found that the STAT3 pathway is abnormally and continuously activated [26]. Additionally, Chen SH et al. compared the immunohistochemical staining of vessel endothelial cells in normal organs and tumor tissues and found that more P-STAT3 was present in tumor tissues than in normal tissues [27]. These results suggest that P-STAT3 regulates the expression of VEGFA and that downregulating the activation of STAT3 can be a potent strategy for inhibiting the overexpressed VEGFA. However, as STAT3 can be activated by multiple cytokines and chemokines [28], a method has not yet been identified to directly suppress the activation of STAT3 in the clinic. An early study demonstrated that the S1P-S1PR1 pathway reciprocally regulates STAT3 activity, which is crucial for malignant progression in cancer cells [29]. The results indicate that S1PR1 positively regulates the activation of STAT3. In addition, research has reported that S1PR1 activity increases tumor growth by amplifying VEGFA angiogenic signaling [30]. These studies suggested that S1PR1 positively affects the expression of VEGFA. We hypothesize that S1PR1 could serve as a potent antitumor and antiangiogenic target in breast cancer by downregulating the P-STAT3/VEGFA pathway.

Siponimod (BAF312), which has been approved by the FDA for treating multiple sclerosis, is a selective antagonist of S1PR1 [31]. A study found that BAF312 (1 h at 1 μM) significantly increases the degradation of S1PR1 by 91%, thereby downregulating the expression of S1PR1 [32]. Early studies suggested that BAF312 inhibits tumor growth and reduces angiogenesis via downregulating S1PR1 expression [33]. Research has found that a first-generation antagonist of S1PRs (S1PR1, S1PR3, S1PR4, and S1PR5), fingolimod (FTY720), inhibits tumor growth and metastasis and increases the chemosensitivity of advanced and hormonal refractory BRCA and TNBC by downregulating S1PR1 [34–36]. These results indicate that BAF312 could be applied to suppress breast tumor growth and inhibit angiogenesis.

We prepared pH-sensitive and tumor-targeted shell-core nanoparticles, in which the hydrophilic PEG2000 chain modified by cyclic Arg-Gly-Asp (cRGD) formed the shell; hydrophobic DSPE formed the core; biodegradable CaP (calcium and phosphate ions) was adsorbed onto the shell. The formed nanoparticles were used as the BAF312 delivery system (BAF312@cRGD-CaP-NPs). The outstanding features of the generated NPs were as follows: (1) The nanoparticles have a diameter of 109.9 ± 1.002 nm, which can utilize the enhanced permeability and retention effect (EPR) to accumulate in the tumor site [37]; (2) cRGD has specific affinity for the αvβ3 and αvβ5 integrins, which are highly expressed on the breast cancer cells and endothelial cells of tumor angiogenic vessels [38, 39]; (3) DSPE is a phospholipid and is harmless to the human body, and PEG2000 is approved by the FDA for usage in the human body [40], which enables the nanoparticle to have long-lasting properties in the blood circulation [41]; the calcium-phosphorus system is sensitive to pH changes, thereby ensuring the accurate drug release of nanoparticles in the acidic tumor microenvironment [42]; (4) The negative charge of − 10.6 ± 0.056 mV of the nanoparticles assists them in evading clearance by immune cells and enables them to have long-lasting circulation; the particles become positively charged at + 6.80 ± 0.013 mV in the acidic tumor niche, ensuring that they are efficiently taken up by tumor cells, as tumors are apt to adsorb positively charged nanoparticles [43, 44]; and (5) In addition, calcium and phosphorus are essential elements for human, while breast cancer patients are prone to calcium deficiency due to hormone abnormalities [45]; thus, calcium and phosphorus can be the supplements for patients with BRCA or TNBC. Above all, pH-sensitive and tumor-targeted nanoparticle is a competent drug delivery system for BAF312 to treat breast cancer.

**Scheme 1:**
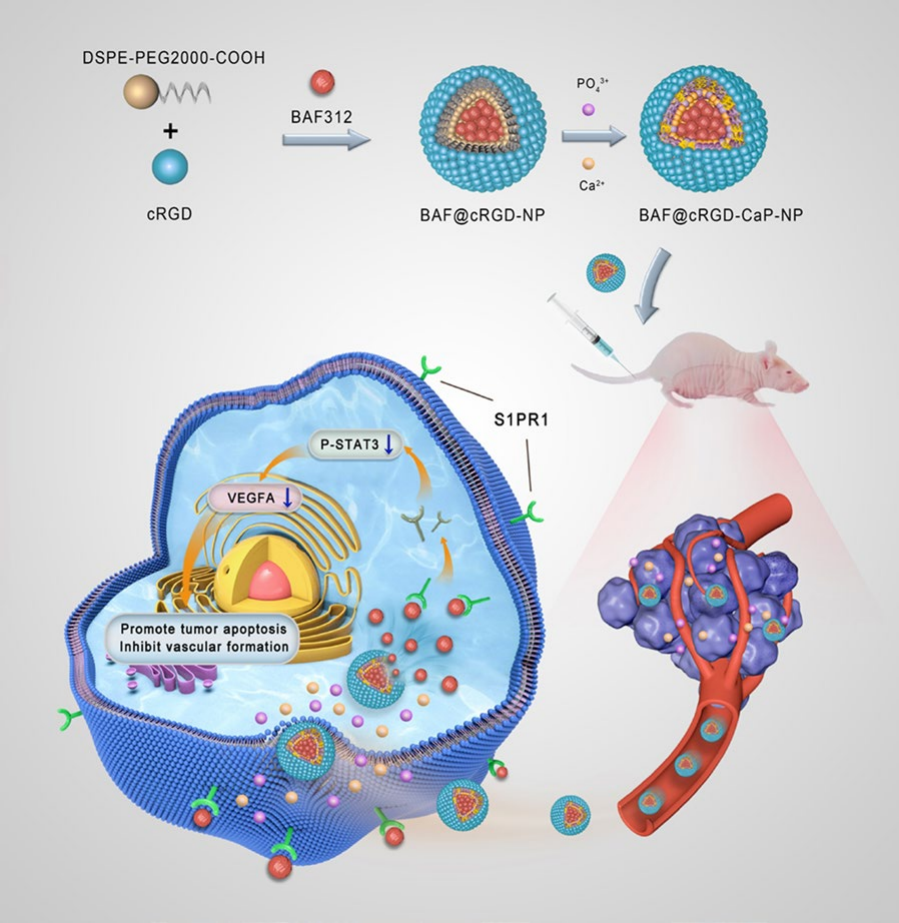
The schematic illustration of BAF312@cRGD-CaP-NPs for tumor treatment through downregulating the S1PR1/P-STAT3/VEGFA axis. The BAF312@cRGD-CaP-NPs are gradually accumulated at the tumor sites in vivo via the EPR effect and active targeting mediated by cRGD and then uptaken by tumor cells and tumor blood vessel cells. Next, the nanoparticles release BAF312 in the acidic tumor microenvironment. Finally, BAF312@cRGD-CaP-NPs induce tumor cell apoptosis and destroy tumor blood vessel to maximize their antitumor efficacy through S1PR1/P-STAT3/VEGFA pathway

In summary, we hypothesize that pH-sensitive and tumor-targeted nanoparticles BAF312@cRGD-CaP-NPs could efficiently inhibit breast tumor growth and angiogenesis by downregulating the S1PR1/P-STAT3/VEGFA axis (Scheme 1), therefore achieving the goal of antitumor growth and inhibiting tumor angiogenesis for patients with BRCA or TNBC.

## Results

### The overexpressed S1PR1 causes the poor survival of patients with breast cancer and has a positive relation with STAT3 and VEGFA

**Fig. 1:**
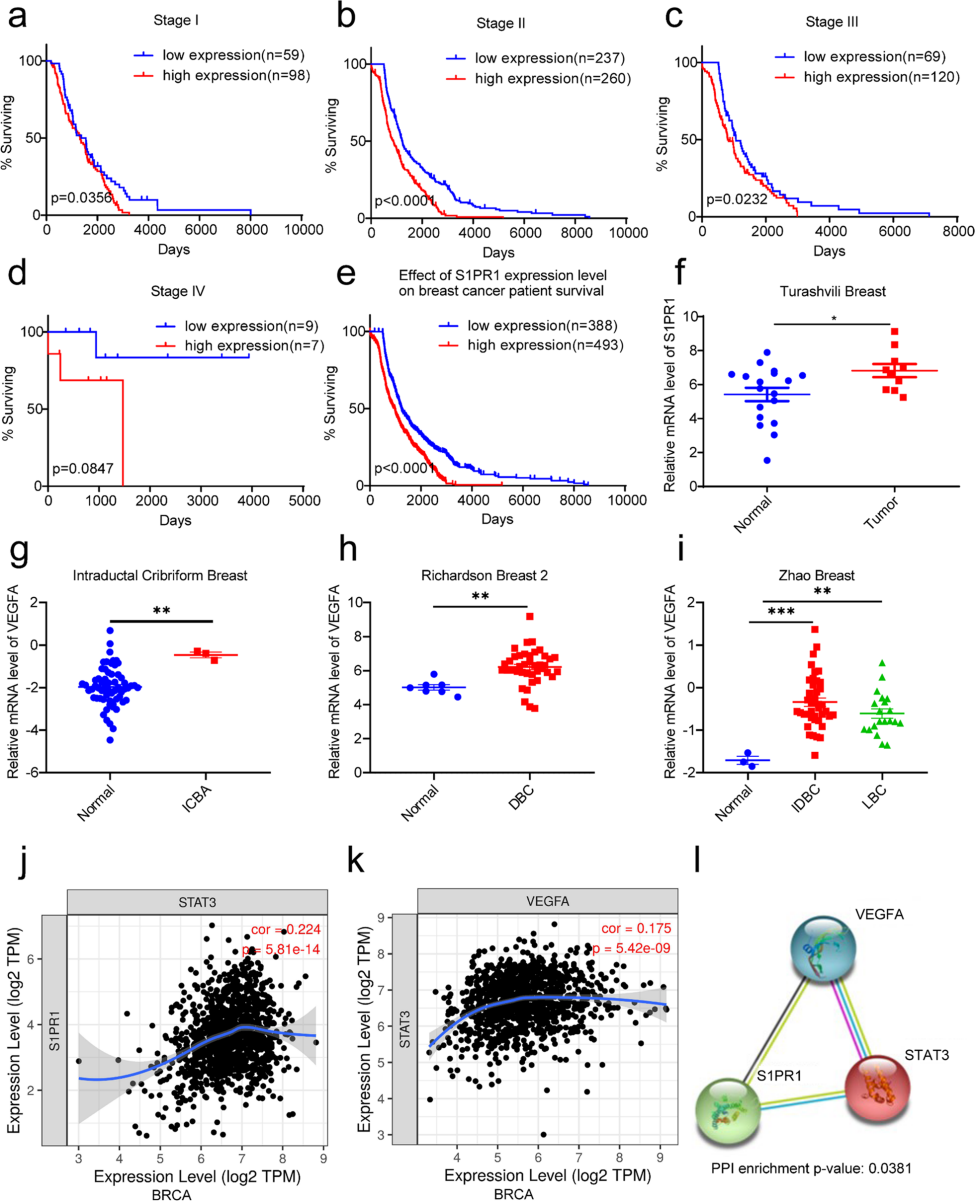
S1PR1 overexpression in BRCA causes poor survival and is positively correlated with STAT3 and VEGFA. **a–e** TCGA database suggest the correlation between patient survival and S1PR1 expression in every stage of breast cancer. **f** Oncomine database analysis of S1PR1 expression in breast cancer and adjacent normal tissue. Mean ± SEM, *P < 0.05. **g–i** Oncomine database analysis of VEGFA expression in breast cancer and adjacent normal tissue. ICBA is intraductal cribriform breast adenocarcinoma, DBC is ductal breast carcinoma, IDBC is invasive ductal breast carcinoma, and LBC is lobular breast carcinoma. Mean ± SEM, **P < 0.01, ***P < 0.001. **j**, **k** TIMER database analysis of the relationship between S1PR1 and STAT3 or STAT3 and VEGFA. “Cor” represents the correlation value, and p is the p value, with values less than 0.05 being considered meaningful. BRCA is breast invasive carcinoma. **l** String database suggest the connection of S1PR1 and STAT3 and VEGFA in breast cancer, p value less than 0.05 is considered meaningful

The TCGA database showed that overexpression of S1PR1 was associated with the poor survival of breast cancer patients in every stage (Fig. 1a–e). Moreover, analysis of the Oncomine database indicated that the S1PR1 expression level was higher in breast cancer than in normal tissues (Fig. 1f), which suggests that S1PR1 could be a novel target for breast cancer treatment. Chang RX et al. analyzed the transcriptome characteristics of patients with TNBC and found that the STAT3 pathway is abnormally and continuously activated [26]. The Oncomine database also showed that the VEGFA expression level was increased in breast cancer (Fig. 1g–i). In addition, the TIMER database showed that S1PR1 and STAT3 had positive connections in breast cancer (Fig. 1j), and STAT3 and VEGFA were positively connected (Fig. 1k). The STRING database showed that S1PR1, STAT3, and VEGFA were connected with each other, and the PPI confidence value was 0.013 (Fig. 1l). These results suggested that S1PR1 overexpression is related to poor survival of patients with breast cancer and is positively correlated with STAT3 and VEGFA.

### S1PR1 affected the progression and chemosensitivity of breast cancer cells and inhibited the vascular formation via regulating the expression of P-STAT3 and VEGFA

**Fig. 2:**
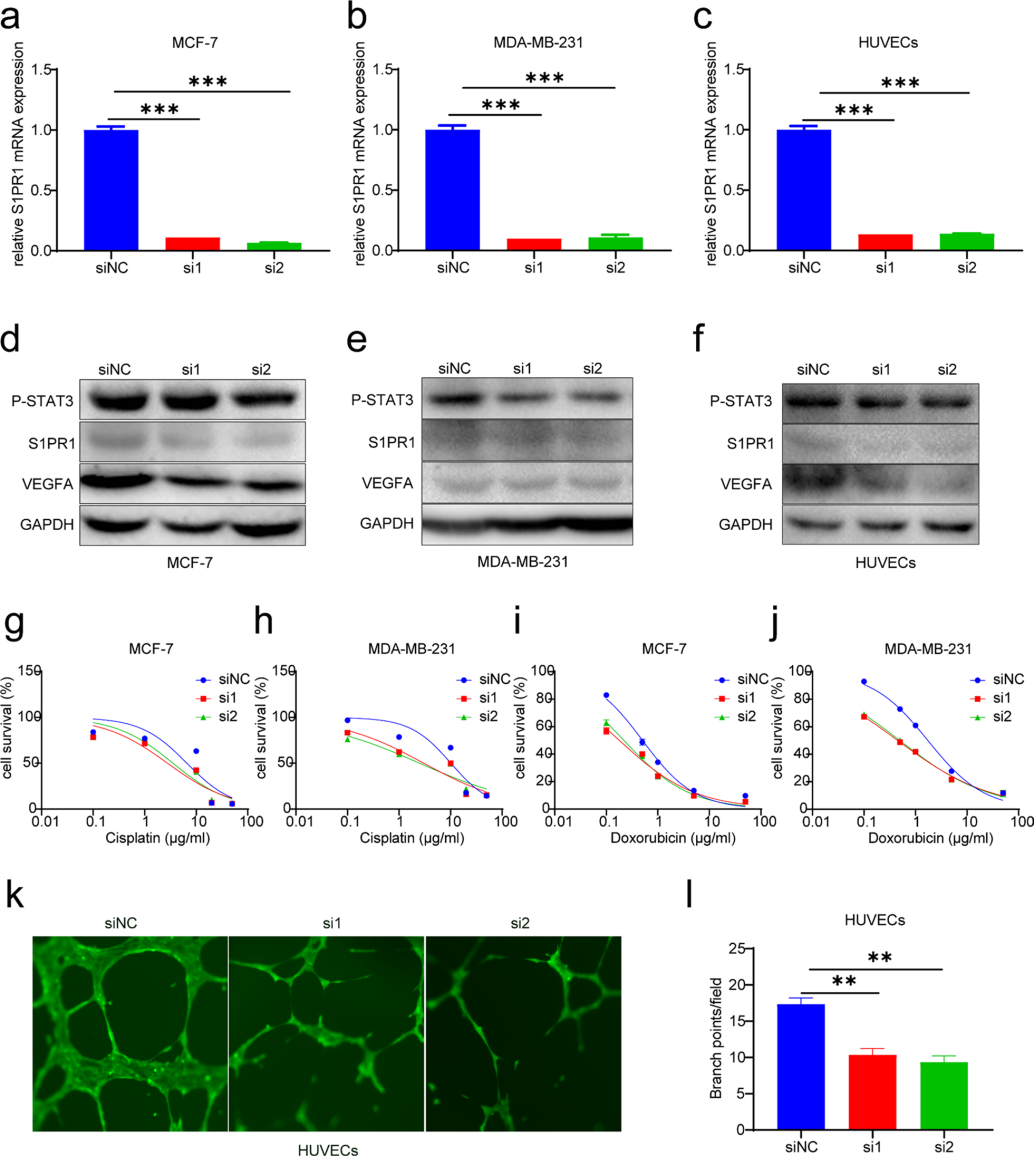
Downregulation of S1PR1 improves the chemosensitivity of BRCA and inhibits vascular formation by decreasing P-STAT3/VEGFA. **a–c** qPCR analysis of S1PR1 expression in MCF-7 and MDA-MB-231 and HUVECs after treated with S1PR1-siRNA for 48 h. Mean ± SEM, ***P < 0.001. **d–f** Western blotting assays show the S1PR1, P-STAT3 and VEGFA protein expression in MCF-7 and MDA-MB-231 cells and HUVECs after treatment with S1PR1-siRNA for 48 h. **g–j** MTT analysis of the viabilities of MCF-7 and MDA-MB-231 cells following treatment with the cisplatin or doxorubicin for 2 days. **k** Vascular formation assay analysis of the tube formation of HUVECs pretreated with S1PR1-siRNA for 20 h. **l** The statistical results show the tube formation number of HUVECs. Mean ± SEM, **P < 0.01

To evaluate the mechanism by which S1PR1 affects the progression of breast cancer, we used small interfering RNA (siRNA) to inhibit the S1PR1 expression in breast cancer cells (MCF-7 and MDA-MB-231) and HUVECs. RT-PCR and western blotting assays suggested that S1PR1 was downregulated by siRNA (Fig. 2a–f). MDA-MB-231 has been confirmed as a malignant tumor cell line and is representative of triple-negative breast cancer (TNBC) cells [46], while MCF-7 is representative of relatively benign tumor cells [47]. Moreover, the CCLE database suggested that the protein expression of S1PR1, STAT3, and VEGFA were higher in MDA-MB-231 than in MCF-7 (Additional file 1: Figure S1). Our study confirmed that MCF-7 cells are more sensitive to doxorubicin (DOX) and cisplatin (DDP) than MDA-MB-231 via using the MTT assay (Additional file 1: Figure S2). Moreover, our study found that downregulating S1PR1 in MCF-7 and MDA-MB-231 cells could restore the chemosensitivity of the two cell types to DOX and DDP (Fig. 2g–j), which suggests that S1PR1 is associated with chemoresistance in breast cancer. We also tested the changes in the proliferation of the two cell lines after altering the expression of S1PR1 and found that the proliferation of the two cell lines was downregulated in the S1PR1-siRNA groups (Additional file 1: Figure S3). These results reveal that downregulating S1PR1 could restore chemosensitivity and downregulate the proliferation of breast cancer cells. Patients with breast cancer, especially TNBC, usually progress rapidly due to the abundant blood supply in tumor sites; hence, it is necessary to interrupt tumor angiogenesis. After downregulating S1PR1 expression, vascular formation was inhibited (Fig. 2k, l), and the proliferation of HUVECs was decreased as well (Additional file 1: Figure S4). Western blotting assays demonstrated that S1PR1 was downregulated, which was followed by P-STAT3 and VEGFA downregulation (Fig. 2d–f). These results suggested that S1PR1 represses tumor growth and inhibits tumor angiogenesis through the S1PR1/P-STAT3/VEGFA axis.

### BAF312 promoted the apoptosis of breast cancer cells and inhibited angiogenesis

**Fig. 3:**
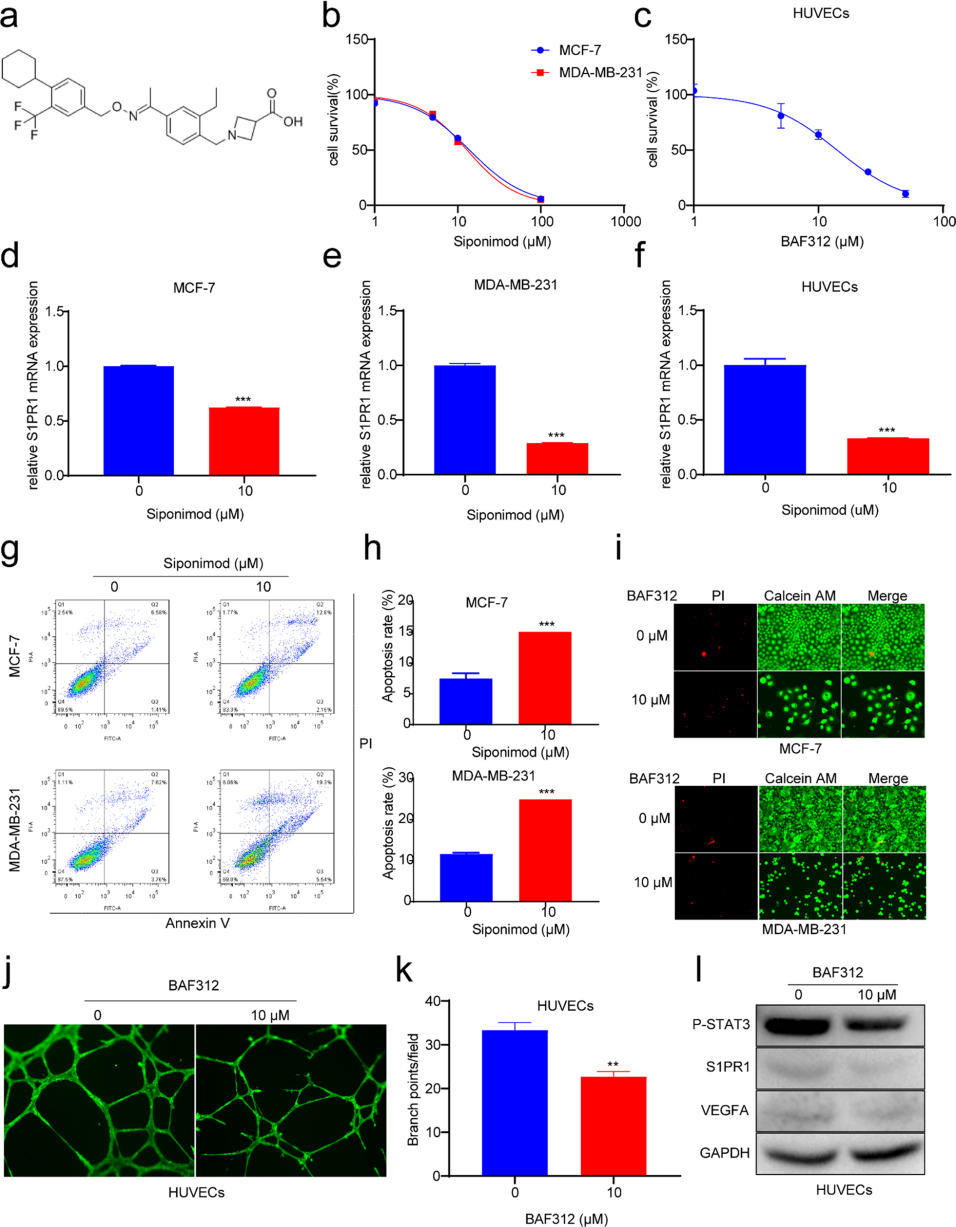
BAF312 promotes apoptosis of BRCA and inhibits vascular formation of HUVECs via downregulating S1PR1/P-STAT3/VEGFA. **a** The structure of BAF312. **b**, **c** MTT assays suggest the cells viability of MCF-7 and MDA-MB-231 and HUVEC following treatment with the BAF312 for 72 h. **d–f** qPCR results show the S1PR1 expression of MCF-7 and MDA-MB-231 and HUVECs after treatment with the BAF312 for 48 h. Mean ± SEM, ***P < 0.001. **g** Apoptosis assays indicate the apoptotic rates of MCF-7 and MDA-MB-231 after treatment with the BAF312 for 48 h. **h** The statistical results show the apoptotic ratio of MCF-7 and MDA-MB-231. Mean ± SEM, ***P < 0.001. **i** Calcein-AM/PI staining analysis of apoptosis rates of MCF-7 and MDA-MB-231 following treatment with 10 µM BAF312 for 72 h. The red fluorescence means PI positivity, and the green fluorescence means calcein positivity. **j** Vascular formation assay analysis of the tube formation of HUVECs incubated with 10 µM BAF312 for 20 h. **k** The statistical results of the tube formation number for HUVECs. Mean ± SEM, **P < 0.01. **l** Western blotting assays show S1PR1 and P-STAT3 and VEGFA expression levels in HUVECs after treatment with 10 µM BAF312 for 48 h

BAF312 was the selected agonist of S1PR1 (Fig. 3a), and we tested the anticancer efficacy of BAF312 in affecting breast cancer cells and HUVECs. The MTT assay suggested that BAF312 could inhibit the progression of both cell lines, and the half-maximal inhibitory concentrations (IC_50_) of BAF312 were almost the same in two cell types (MCF-7: 13.86 μM, MDA-MB-231: 12.96 μM) (Fig. 3b), which suggested that BAF312 could kill these two types of tumor cells equally. We also tested the inhibitory effect of BAF312 on HUVECs. The MTT assay showed that BAF312 could inhibit the proliferation of HUVECs, and the IC_50_ was 14.11 μM (Fig. 3c). RT-PCR suggested that BAF312 downregulated S1PR1 mRNA levels in breast cancer cells and HUVECs (Fig. 3d–f), and Western blotting assays indicated that BAF312 decreased the level of S1PR1 at the protein level (Fig. 3l). Flow cytometry suggested that BAF312 improved the apoptosis rate of MCF-7 and MDA-MB-231 cells (Fig. 3g, h), and PI and calcein-AM staining also confirmed that BAF312 increased the apoptosis of the two cell lines (Fig. 3i). Simultaneously, we found that BAF312 could effectively decrease the vascular formation of HUVECs (Fig. 3j, k). Western blotting assays confirmed that BAF312 could downregulate the expression of S1PR1, P-STAT3, and VEGFA (Fig. 3l). These results supported that BAF312 could inhibit the survival rate of breast cancer by promoting apoptosis of breast cancer cells and decreasing vascular formation of HUVECs via S1PR1/P-STAT3/VEGFA axis.

### Formation of nanoparticle BAF312@cRGD-CaP-NPs and testing of their features

**Fig. 4:**
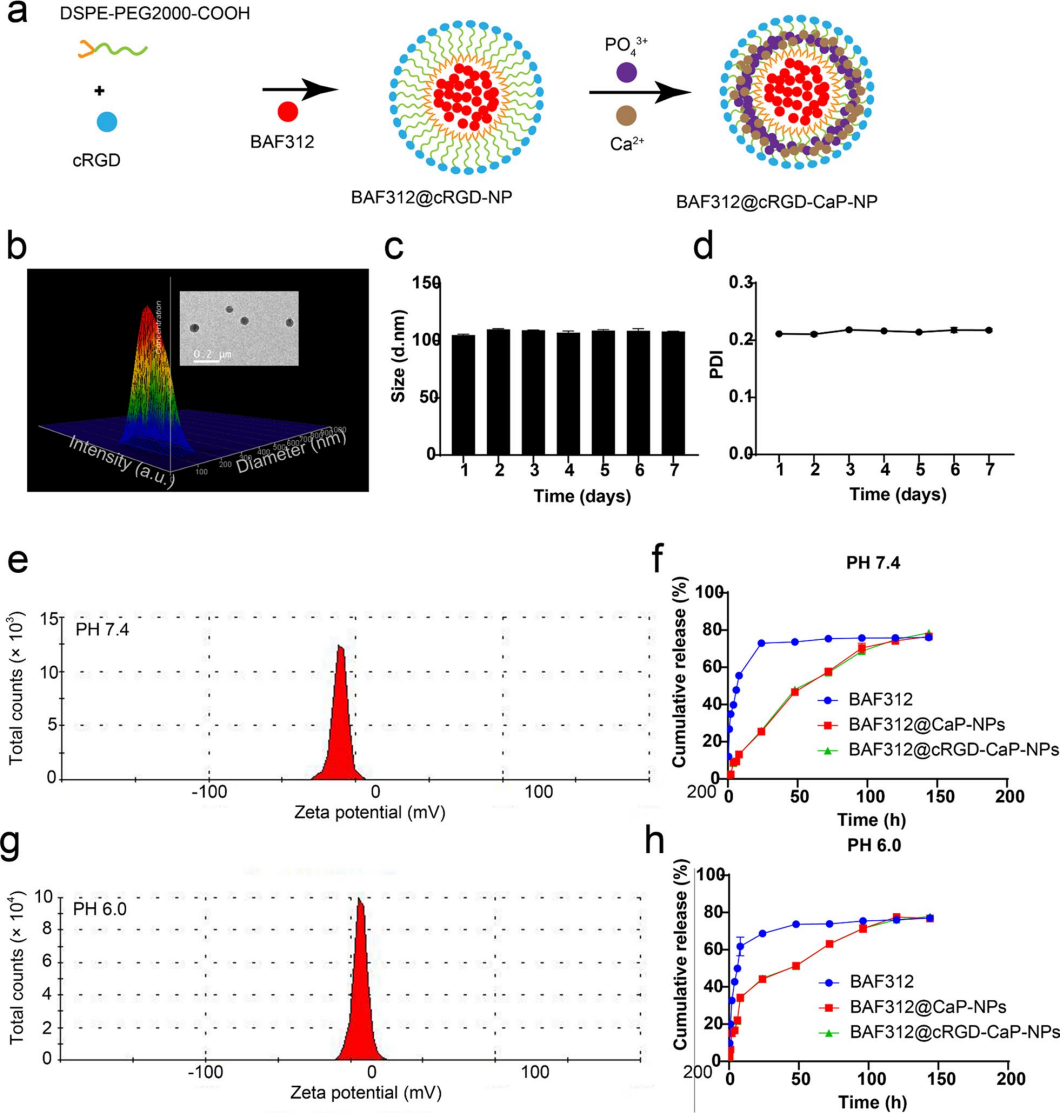
Formation process and characterization of pH-sensitive shell–core BAF312@cRGD-CaP-NPs. **a** Schematic diagram of the BAF312@cRGD-CaP-NP formation process. **b** Nanosize indicated that the BAF312@cRGD-CaP-NP size was 109.9 ± 1.002 nm, with a PDI of 0.253 ± 0.006. TEM results indicated BAF312@cRGD-CaP-NPs were spherical and homogeneous. **c**, **d** The stability of BAF312@cRGD-CaP-NPs were tested for 7 days. **e**, **g** The average potentials of BAF312@cRGD-CaP-NPs in aqueous solutions of pH 7.4 and pH 6.0 were − 10.6 ± 0.056 mV and + 6.80 ± 0.013 mV, respectively. **f**, **h** HPLC assay showed the BAF312 release profiles from NPs or NPs modified with cRGD at pH 7.4 and 6.0

The biomineralization method were adopted to form the nanoparticles BAF312@cRGD-CaP-NPs of which the DSPE and BAF312 formed the inner core and the PEG2000 modified with cRGD absorbing calcium-phosphate formed the outer shell (Fig. 4a and Additional file 1: Figure S5). TEM results suggested that BAF312@cRGD-CaP-NPs were spherical and distributed uniformly (Fig. 4b), and had the diameter of 109.9 ± 1.002 nm with the PDI of 0.253 ± 0.006 (Fig. 4b). The size changes of NPs after incubated at 37 °C in 5% serum solution (pH 7.4) was recorded to measure the stability. There is nearly no change of size and PDI of NPs for 7 days, which suggest the NPs could keep stable in the circulation (Fig. 4c, d). CaP was sensitive to pH changes and assured the accurate release of BAF312 in the tumor acidic microenvironment. In vitro, we simulated the internal drugs release by dialysis analysis, which suggested that BAF312 was released from NPs faster at pH 6.0 than at pH 7.4 (Fig. 4f, h). The zeta potential of NPs was − 10.6 ± 0.056 mV at pH 7.4 (Fig. 4e). However, the zeta potential of NPs was + 6.80 ± 0.013 mV at pH 6.0 (Fig. 4g). Additional file 1: Figure S6 indicated that cRGD-modified nanoparticle can exist in the blood circulation for a longer time than the free drug group, combined with a previous study, the positive potential enabled the NPs to be free to travel through the blood circulation, and the negative potential of NPs ensured that they were effectively absorbed by tumor cells, which suggested that the features of NPs were enhancing absorption by tumor cells. Moreover, the drug loading (DL%) value and the encapsulation efficiency (EE%) value for BAF312 were 13.57% and 81.4%, respectively. These relatively high DL% values and high EE% values could ensure that BAF312@cRGD-CaP-NPs have fewer side effects and higher therapeutic effects.

### Nanoparticle BAF312@cRGD-CaP-NPs enhanced the uptake of breast cancer cells and HUVECs and lysosome escape

**Fig. 5:**
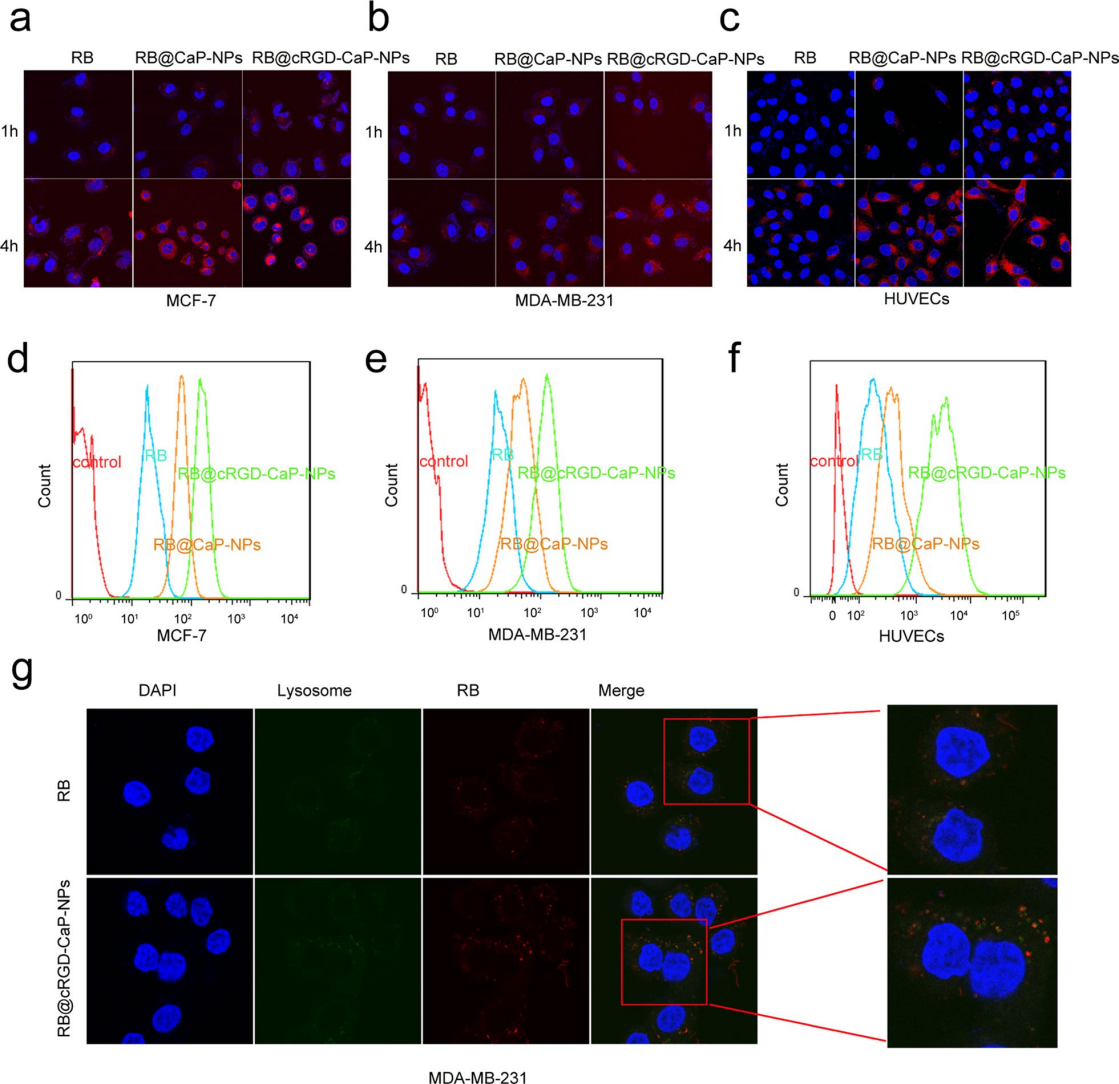
NPs modified with cRGD enhanced BRCA and HUVECs uptake of NPs and lysosomal escape occurred. **a–c** The cellular uptake of free RB and RB@CaP-NPs and RB@cRGD-CaP-NPs in MCF-7 and MDA-MB-231 and HUVECs were detected by the fluorescence microscope. **d–f** Flow cytometry assays show the fluorescence intensities of RB and RB@CaP-NPs and RB@cRGD-CaP-NPs in cells for 4 h. **g** Blue fluorescence indicates the nucleus; Red fluorescence indicates the RB or RB@cRGD-CaP-NPs; Green fluorescence indicates the lysosome (LysoTracker); The overlap of green fluorescence and red fluorescence indicate that most free RBs were entrapped in the lysosome, while the green and red fluorescence separately existed in the NPs group indicated that most NPs were successfully escaped from lysosomes

The effective internalization of drugs by cells is a prerequisite for drug therapy to achieve antitumor effects; thus, we carried out a fluorescence microscopy assay, which could effectively record the transportation process of nanoparticles into breast cancer cells. We used the fluorescent dye RB to replace BAF312 to develop RB@CaP-NPs and RB@cRGD-CaP-NPs to record the NPs in breast cancer cells and HUVECs. We observed the fluorescence density under an inverted fluorescence microscope: RB < RB@CaP-NPs < RB@cRGD-CaP-NPs, indicating that NPs modified with cRGD greatly promote the uptake rate of the drugs (Fig. 5a–c). Moreover, the flow cytometry assay also suggested that the absorption of RB@cRGD-CaP-NPs was greater than that of the other groups (Fig. 5d–f). Besides, Additional file 1: Figure S7 suggested that BAF312@cRGD-CaP-NPs and BAF312@CaP-NPs also escape from the immune cells. These results indicated that the nanoparticles were highly effective drug carriers. An important step for successful drug delivery is that the intracellular NPs should be transported from the lysosome to the cytoplasm. Figure 5g showed that most free drugs confined in lysosomes in the RB group and most NPs successfully escaped the lysosomes in the NPs group, because there existed many overlaps of green and red fluorescence in RB group, while there had lots of obvious separation images of green and red fluorescence in the NPs group. Therefore, NPs can evade lysosome degradation and then increase the amounts of drugs that enter the cytoplasm to further exert their effects.

### *BAF312@cRGD-CaP-NPs effectively promoted apoptosis of breast cancer cells and inhibited vascular formation of HUVECs *via* the S1PR1/P-STAT3/VEGFA axis*

**Fig. 6:**
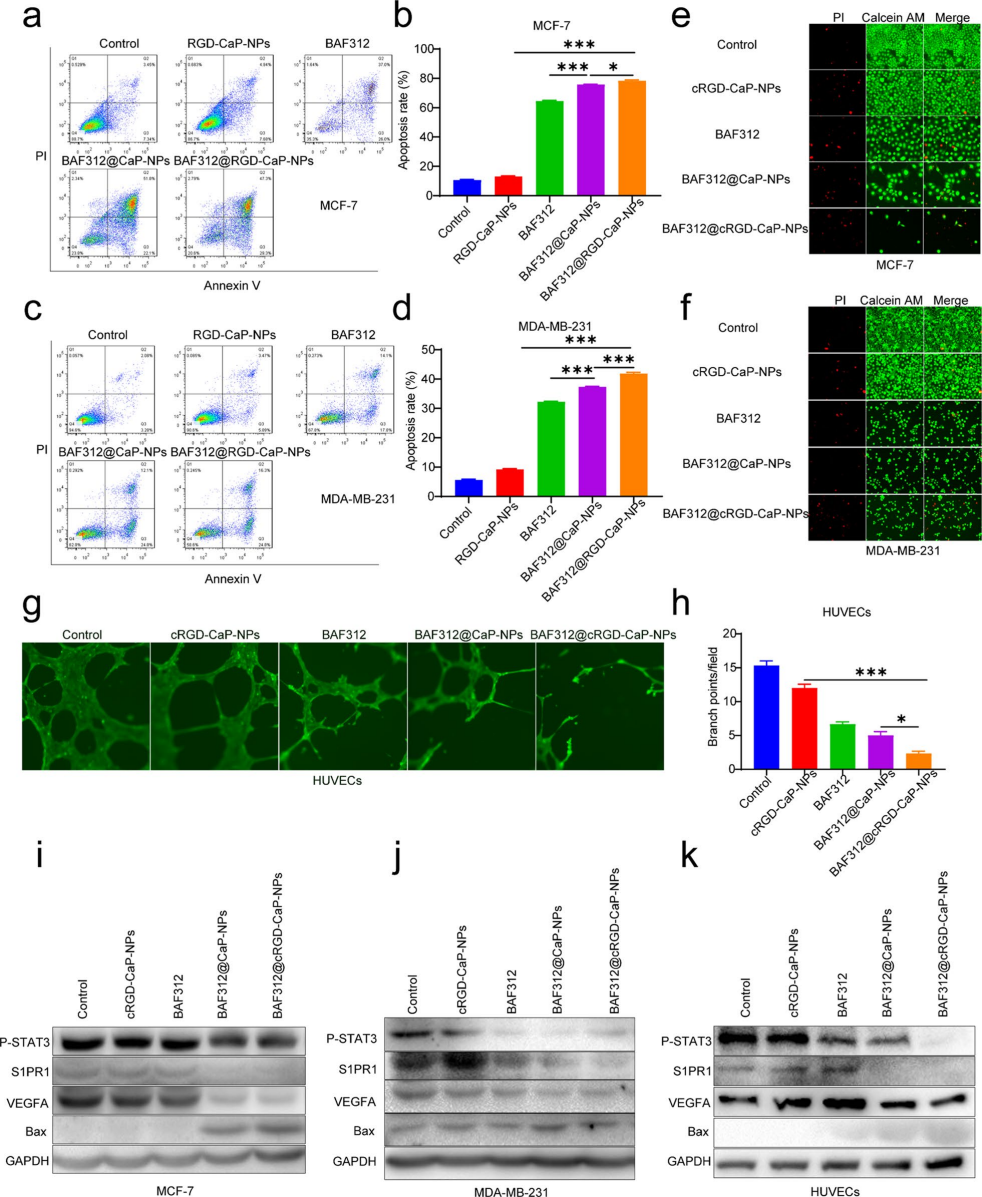
BAF312@cRGD-CaP-NPs boost the apoptosis of BRCA and inhibit the vascular formation via downregulating S1PR1/P-STAT3/VEGFA. **a**, **c** The apoptotic ratio of MCF-7 and MDA-MB-231 cells were detected by the apoptosis assay after treatment with 10 µM BAF312, 10 µM BAF312@CaP-NPs, and 10 µM BAF312@cRGD-CaP-NPs for 2 days. **b**, **d** The statistical results revealed the apoptotic ratio for cells. Mean ± SEM, *P < 0.05, *** P < 0.001. **e**, **f** Calcein-AM/PI staining indicated the apoptosis of MCF-7 and MDA-MB-231 cells after treatment with 10 µM BAF312, 10 µM BAF312@CaP-NPs, and 10 µM BAF312@cRGD-CaP-NPs for 3 days. The red fluorescence means PI positivity, and the green fluorescence means calcium positivity. **g** Vascular formation assay analysis of the tube formation of HUVECs after treatment with 10 µM BAF312, 10 µM BAF312@CaP-NPs, and 10 µM BAF312@cRGD-CaP-NPs for 20 h. **h** The statistical results showed tube formation rates for HUVECs. Mean ± SEM, * P < 0.05, ***P < 0.001. **i–k** Western blot analyzed proteins expression of S1PR1, P-STAT3, VEGFA, and Bcl-2 in breast cancer cells and HUVECs after treatment with 10 µM BAF312, 10 µM BAF312@CaP-NPs, and 10 µM BAF312@cRGD-CaP-NPs for 2 days

Previous researches have suggested that BAF312 inhibits the proliferation of breast cancer cells by promoting their apoptosis; hence, we used apoptosis assays to assess the effects of BAF312@cRGD-CaP-NPs on breast cancer cells (MCF-7 and MDA-MB-231). The final results demonstrated that the apoptotic rates of two kinds of breast cancer cells were as follows: control group < cRGD-CaP-NPs group < BAF312 group < BAF312@CaP-NPs group < BAF312@cRGD-CaP-NPs goup (Fig. 6a–d). The Calcium-AM/PI double staining assays also demonstrated that the apoptosis rate induced by BAF312@cRGD-CaP-NPs was much higher than that of the other groups (Fig. 6e, f). Simultaneously, we assessed the effect of BAF312@cRGD-CaP-NPs on the tube-forming ability and migration ability of HUVECs. The results demonstrated that nanoparticles effectively reduced the vascularization ability of HUVECs (Fig. 6g, h and Additional file 1: Figure S8). Western blot assays demonstrated that nanoparticles achieve antitumor growth and antivascular formation effects by downregulating the expression of S1PR1, P-STAT3 and VEGFA (Fig. 6i–k). In addition, we found that BAF312@cRGD-CaP-NPs could effectively inhibit the migration of MCF-7 and MDA-MB-231 cells (Additional file 1: Figure S9), which indicated that BAF312 might become the candidate for repressing the tumor metastasis of patients with BRCA, especially TNBC.

### Nanoparticles modified with cRGD effectively targeted the tumors in vivo

**Fig. 7:**
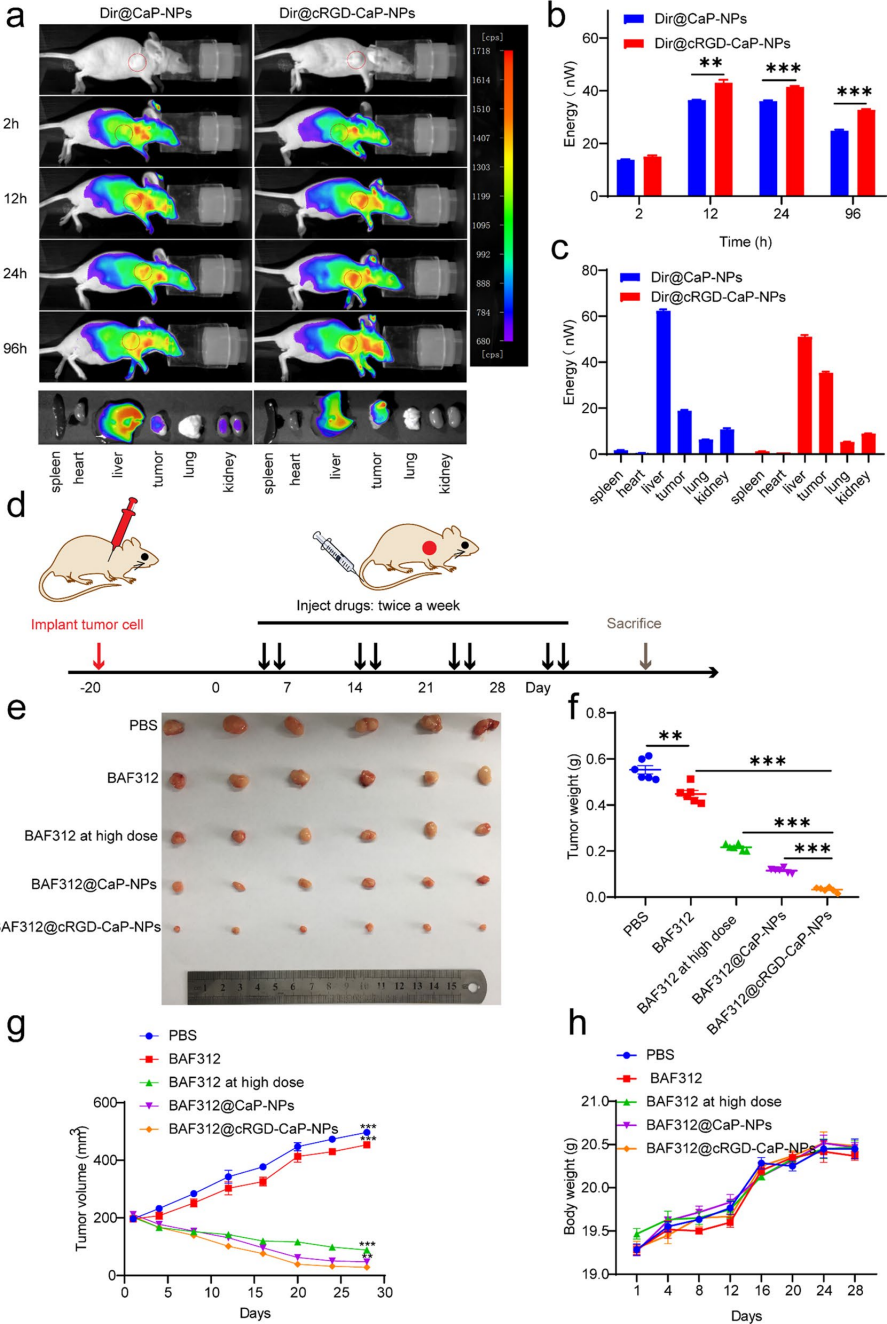
The tumor targeting ability of cRGD-NPs and the antitumor efficiency of BAF312@cRGD-CaP-NPs in vivo. **a** Upper: After intravenous injection for 2, 12, 24 and 96 h, the distribution of Dir@CaP-NPs and Dir@cRGD-CaP-NPs in MDA-MB-231 tumor-bearing nude mice (Dir dose is 1 μg/mL) (n = 3). Below: Fluorescence images of anatomic tumors and other major organs (spleen, heart, liver, lung, and kidney) at 96 h after the intravenous injection of Dir@CaP-NPs and Dir@cRGD-CaP-NPs. **b** Quantitative analysis of the mean Dir fluorescence intensity of tumor sites of intravenous injection of Dir@CaP-NPs and Dir@cRGD-CaP-NPs for 2, 12, 24, and 96 h. Mean ± SEM, n = 3, **P < 0.01, ***P < 0.001. **c** Quantitative analysis of the mean Dir fluorescence intensity of the anatomic tumors and major organs (spleen, heart, liver, lung, and kidney) at 96 h after the intravenous injection of Dir@CaP-NPs and Dir@cRGD-CaP-NPs. **d** Schematic illustration of the MDA-MB-231 tumor implantation and the treatments in nude mice. Twenty days after tumor implantation, these mice were treated with the indicated treatments on the indicated days. **e** Fig. of tumors collected from the different treatment groups. **f** The tumor weights collected from different groups after anatomy (n = 6). **g** The tumor growth curves. **h** The changes of body weights

The successful anticancer drug delivery systems should own the abilities of accumulation and penetration into the tumor sites. Hence, the biodistribution of NPs in vivo was checked. We used the fluorescent dye Dir to replace BAF312 to develop Dir@CaP-NPs and Dir@cRGD-CaP-NPs to track the in vivo distribution of NPs. MDA-MB-231 tumor-bearing BALB∕c nude mice were injected with Dir@CaP-NPs or Dir@cRGD-CaP-NPs at a 0.5 mg kg^−1^ Dir dosage intravenously. Time-dependent fluorescence biodistributions were recorded with the in vivo imaging system (Fig. 7a). The fluorescence signals of Dir@CaP-NPs was mainly enriched in the liver, and few of them was enriched in the tumor site at 2 h, increased to a maximum at 12 h, and then gradually decreased. However, the fluorescence signals of the Dir@cRGD-CaP-NP groups were primarily enriched at the tumor at 12 h and gradually increased in the tumor as time up to 24 h (Fig. 7b). After injection for 96 h, the tumors and other major organs (heart, liver, spleen, lung and kidney) were collected for measuring fluorescence biodistributions. The tumor fluorescence intensities of the Dir@cRGD-CaP-NP group were much stronger than the Dir@CaP-NP group (Fig. 7c), indicating that nanoparticles modified with cRGD greatly increased drug accumulation and penetration in the tumor site and improved the usage of drugs. These data demonstrated that Dir@CaP-NPs could passively accumulate and penetrate into the tumor sites via the EPR effect, while the aggregation of Dir@cRGD-CaP-NPs at the tumor site depends not only on the EPR effect but also on the specific recognition of tumors by cRGD. The prolonged circulation characteristics of nanoparticles modified with cRGD may show protection against hepatic phagocytosis, and it is beneficial to passively target tumors through the EPR effect. Compared with Dir@CaP-NPs, Dir@cRGD-CaP-NPs showed significantly better tumor selectivity (Fig. 7c). These results suggested that NPs of which modificated with cRGD ligand improves the accumulation and penetration of NPs in tumor tissues via active targeting.

### Excellent anti-cancer and anti-angiogenesis activity of BAF312@cRGD-CaP-NPs in vivo

**Fig. 8:**
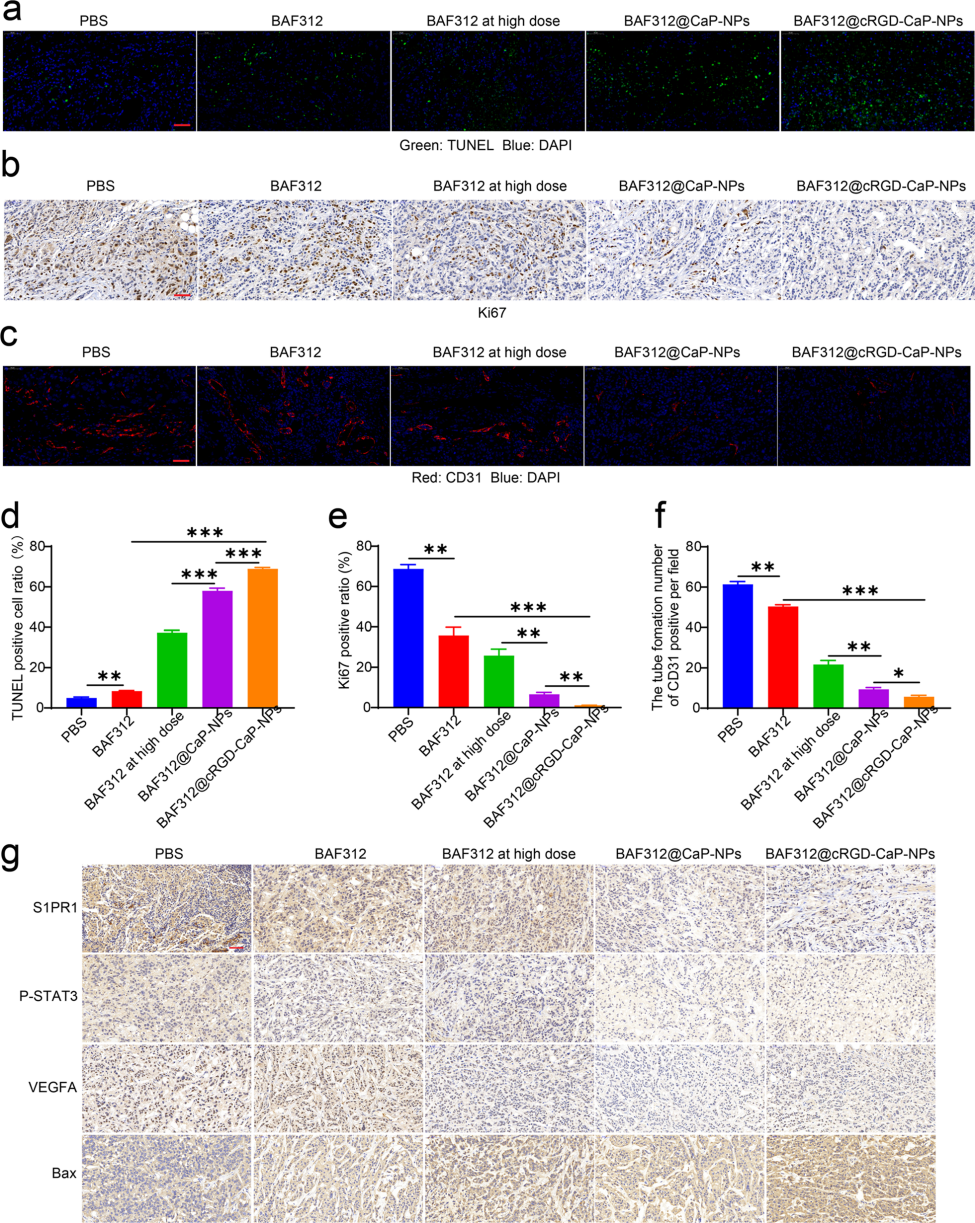
BAF312@cRGD-CaP-NPs inhibit tumor growth and angiogenesis in vivo via downregulating the expression of the S1PR1/P-STAT3/VEGFA. **a** Immunofluorescence assay analysis of TUNEL in the tumors. Scale bar: 50 µm. **b** IHC analysis of Ki67 expression in the tumors. Scale bar: 50 µm. **c** Immunofluorescence assay analysis of CD31 in the tumors. Scale bar: 50 µm. **d** The statistical results of TUNEL positivity in MDA-MB-231 tumors. Mean ± SEM, n = 3, **P < 0.01, ***P < 0.001. **e** The statistical results of Ki67 positivity in MDA-MB-231 tumors. Mean ± SEM, n = 3, **P < 0.01, ***P < 0.001. **f** The statistical results of CD31 for MDA-MB-231 tumors. Mean ± SEM, n = 3, *P < 0.05, **P < 0.01, ***P < 0.001. **g** The proteins expression of S1PR1, P-STAT3, VEGFA, and Bax were determined by IHC in tumors of which collected at the end point. Scale bar: 50 µm

The in vivo anticancer activity of BAF312@cRGD-CaP-NP was determined in BALB/c nude mice bearing subcutaneous MDA-MB-231 tumors. BAF312 (5 mg kg^−1^), BAF312 at a high dose (10 mg kg^−1^), BAF312@CaP-NPs, and BAF312@cRGD-CaP-NPs at a BAF312 dosage of 5 mg kg^−1^ were injected to MDA-MB-231 tumor-bearing mice intravenously twice a week for 28 days (Fig. 7d). The tumors grew very fast, and BAF312 (5 mg kg^−1^) and BAF312 at a high dose (10 mg kg^−1^) and BAF312@CaP-NPs at a BAF312 dosage of 5 mg kg^−1^ did not significantly repress tumor growth compared with PBS group (Fig. 7e–g). In contrast, BAF312@cRGD-CaP-NPs at a BAF312 dosage of 5 mg kg^−1^ indicated significant anticancer activity and were even stronger than free BAF312 at a high dosage (10 mg kg^−1^) (Fig. 7e–g). The increased TUNEL-positive apoptotic tumor cells and decreased Ki67-positive proliferation tumor cells in excised tumor tissues were further confirmed the excellent anticancer activity of BAF312@cRGD-CaP-NPs (Fig. 8a, b, d, e).

To further verify whether BAF312@cRGD-CaP-NPs could efficiently inhibit tumor neovascularization, the tumor tissues after treatment were stained with CD31. CD31-positive cells were significantly inhibited in the BAF312@cRGD-CaP-NP-treated group compared with the BAF312-, BAF312 at high dose-, or BAF312@CaP-NP-treated groups (Fig. 8c, f). To further confirm the underlying mechanism by which BAF312 inhibits tumor growth and antivascular formation, we conducted immunohistochemical analysis, and the results indicated that S1PR1, P-STAT3, and VEGFA were downregulated. In addition, the expression of Bax was increased, which further suggested that BAF312 promotes apoptosis (Fig. 8g). Importantly, there was no change in body weight (Fig. 7h) and hematoxylin–eosin (H&E) staining of major organs (Additional file 1: Figure S10) indicating that BAF312@cRGD-CaP-NPs did not cause systemic toxicity in mice. Moreover, NPs could protect the mice from fractures (Additional file 1: Figure S11). Taken together, these results strongly suggest that BAF312@cRGD-CaP-NPs have excellent anticancer activity and inhibit neovascularization by downregulating S1PR1/P-STAT3/VEGFA signaling.

## Discussion

Our results have several implications. More efficacious hormone- and chemo-based therapeutic strategies are associated with treatment resistance or secondary malignancy in patients with BRCA, especially TNBC [48, 49]. A study found that the continuous activation of STAT3 and the overexpression of VEGFA occur with high frequency in breast cancer patients and contribute to chemoresistance and secondary malignancy [12–15]. Persistent and abnormal activation of STAT3 has always been linked to malignant cancer behaviors, including proliferation, metastasis, and invasion [26]. The phosphorylation of STAT3 regulates the activity of STAT3 [50]. There are no available effective drugs for regulating the phosphorylation of STAT3 in the clinic; thus, finding the upstream regulator of P-STAT3 seems to be important. VEGFA is one of the most potent mediators of angiogenesis and promotes unrestricted tumor growth [16]. Hence, there is an urgent need to identify potent targets to suppress the activation of STAT3 and overexpression of VEGFA.

A previous study confirmed that S1PR1 is crucial for persistent STAT3 activation in cancer [29]. Aberrant S1PR1/P-STAT3 signaling loops have been detected in various tumor types [29]. These studies indicated that inhibiting the expression of S1PR1 might be broadly effective for downregulating P-STAT3. In addition, S1PR1 overexpression has been shown to promote breast tumor growth and angiogenesis [7]. A study confirmed that downregulating S1PR1 could affect the activity of the VEGFA pathway [30]. Moreover, research has found that activated STAT3 enhances the expression of VEGFA and then promotes the growth of the tumor vasculature [27]. A previous study applied chromatin immunoprecipitation assays to reveal that the STAT3 protein binds to the VEGFA promoter in vivo [24] and found that constitutive STAT3 activity upregulates VEGFA expression and increases tumor angiogenesis [51]. All these findings suggested that S1PR1 affects tumor growth by affecting P-STAT3/VEGFA signaling.

Here, we determined that the level of S1PR1 in breast cancer cells is positively correlated with STAT3 activation and VEGFA expression. According to some reports, MDA-MB-231 cells are TNBC cells [46], while MCF-7 cells are relatively benign BRCA cells[47]. Additional file 1: Figure S2 suggests that MDA-MB-231 cells are more resistant to DDP and DOX than MCF-7 cells. Moreover, the CCLE database indicated that the expression of S1PR1 in MDA-MB-231 cells was higher than that in MCF-7 cells (Additional file 1: Figure S1). We also found that downregulating S1PR1 using siRNA dramatically restored the chemosensitivity of MCF-7 and MDA-MB-231 cells to DDP and DOX. Western blotting analysis suggested that the decrease in S1PR1 was followed by the downregulation of P-STAT3 and VEGFA, in accordance with the result from CCLE showing that the protein expression of STAT3 and VEGFA was lower in MCF-7 cells than in MDA-MB-231 cells. These results indicate that S1PR1 is associated with chemoresistance by regulating P-STAT3 and VEGFA. Additionally, our work shows that decreasing the expression of S1PR1 using the selective antagonist BAF312 dramatically inhibits STAT3 activity and promotes apoptosis in breast cancer cells, which is consistent with previous research results showing that S1PR1 could help activate STAT3 and that activated STAT3 greatly elevates the expression of VEGF and suppresses the expression of Bax to evade apoptosis. Moreover, we found that downregulating S1PR1 using S1PR1-siRNA or BAF312 could effectively decrease the vascular formation of HUVECs by downregulating P-STAT3/VEGFA signaling. All these results suggest that S1PR1 affects tumor growth and vascular formation through the S1PR1/P-STAT3/VEGFA pathway. For the first time, our work confirms that S1PR1 affects tumor growth and angiogenesis via the S1PR1/P-STAT3/VEGFA axis.

BAF312 is a fat-soluble drug; therefore, we encapsulated BAF312 into pH-sensitive and tumor-targeted nanoparticles to form the BAF312@cRGD-CaP-NPs, of which constructed with a PEG2000 hydrophilic chain modified with cRGD, a DSPE hydrophobic chain and a degradable calcium-phosphate shell. The features of the shell-core structure NPs, including the proper size, the αvβ3 and αvβ5 integrin receptor cRGD and negative charge, ensure their effective penetration of discontinuous tumor blood vessels via EPR effects and active targeting to tumor sites mediated by cRGD and long-term circulation [37, 38, 43]. In addition, the calcium-phosphorus system is sensitive to pH changes, which ensures that the particles become positively charged in the acidic tumor microenvironment [42] and increases the absorption of NPs by tumor cells and vascular cells [44]. In conclusion, the nanoparticles are biocompatible and cause low toxicity and low immunogenicity, which would not lead to an inflammation storm. Moveover, using the NPs delivery system could evade the inherent toxicity of drugs of the dose limitation that occur when they travel through the blood [52]. In vitro and in vivo studies both confirm that BAF312@cRGD-CaP-NPs showed dramatically improved tumor targeting ability and can effectively improve the antitumor effect of BAF312 and inhibit tumor angiogenesis, which is mediated through the S1PR1/P-STAT3/VEGFA pathway. Besides, our research shows that BAF312@cRGD-CaP-NPs can effectively inhibit the metastasis of breast tumor cells, and previous studies have shown that BAF312 can overcome the blood–brain barrier and enter the brain to exert its effects [53]. These results provide new insights that suggest that BAF312@cRGD-CaP-NPs can be used as an effective drug for treating the brain metastasis of breast cancer and deserve further evaluation for the treatment of metastatic breast cancer.

## Conclusions

In summary, our work demonstrates that suppressing S1PR1 could inhibit the malignant progression of breast cancer, and the regulatory effect is achieved by regulating the S1PR1/P-STAT3/VEGFA pathway. The coregulation of both tumor cells and vascular cells has profound biological and therapeutic implications for BRCA as well as TNBC. In addition, the S1PR1 antagonist BAF312 effectively inhibited tumor growth and decreased angiogenesis by affecting S1PR1/P-STAT3/VEGFA signaling. The pH-sensitive and tumor-targeted nanoparticle BAF312@cRGD-CaP-NP shows greatly improved antitumor efficacy and suppression of angiogenesis via the S1PR1/P-STAT3/VEGFA axis in BRCA, especially in TNBC.

## Methods

### Cell culture

We purchased the human breast cancer cell lines MCF-7 and MDA-MB-231 and human umbilical vein endothelial cells (HUVECs) and mouse macrophage cell line RAW246.7 from the China Academy of Sciences and kept them in DMEM (Fisher, USA) medium of which added with 1 × penicillin–streptomycin (Fisher, USA) and 10% fetal bovine serum (Fisher, USA) at 37 °C in a humidified 5% CO_2_ standing-temperature incubator.

### Preparation of nanoparticles

We purchased the 1,2-Distearoyl-*sn*-glycero-3-phosphoethanolamine-*N*-[carboxy(polyethylene glycol)-2000] (DSPE-PEG2000-COOH) and 1,2-dipalmitoyl-*sn*-glycero-3-phosphoethanolamine-*N*-(polyethylene glycol)-c(RGDyk) (DSPE-PEG2000-cRGD) from Ponsure Biotechnology (Shanghai, China). BAF312 was obtained from Selleck (Shanghai, China), and other biological reagents were obtained from Sigma-Aldrich (USA). BAF312-loaded DSPE-PEG2000 or DSPE-PEG2000-cRGD particles were developed via the thin-membrane hydration method. Subsequently, add the CaCl_2_ solution into the prepared particle solution. After that, quickly mix the HBS (Hepes, Na3PO4, and NaCl; pH 7.4) buffer solution with the prepared BAF312-DSPE-PEG2000-Ca^2+^ or BAF312-DSPE-PEG2000-cRGD-Ca^2+^ solution and keep them still for 30 min at room temperature to develop the BAF312@CaP-NPs or BAF312@cRGD-CaP-NPs. To carry out some other experiments, BAF312 was replaced by Dir (Biotium, USA) or rhodamine B (RB) (Beyotime, China) to prepare Dir@CaP-NPs and Dir@cRGD-CaP-NPs or RB@CaP-NPs and RB@cRGD-CaP-NPs, respectively.

### Measurement of chemical and physical properties of nanoparticles

BAF312@cRGD-CaP-NPs were measured with the Zetasizer IV analyzer (Malvern Zetasizer Nano ZS90, Malvern, U.K.) to learn their size and surface potential. BAF312@cRGD-CaP-NPs were scanned with a Talos F200X transmission electron microscope (TEM) to confirm the morphology. The high-performance liquid chromatography (HPLC) (Agilent 1100, USA) was applied to evaluate the drug loading (DL%) and encapsulation efficiency (EE%) of BAF312 in BAF312@cRGD-CaP-NPs.

### In vitro BAF312 release from nanoparticles

A dialysis assay was performed to confirm the BAF312 release profile from BAF312@CaP-NPs or BAF312@cRGD-CaP-NPs in vitro. The solution containing nanoparticles was dialyzed against phosphate-buffered saline (PBS) (Servicebio, China) buffer solution of which added with 10 M sodium salicylate at pH 6.0 or pH 7.4, respectively.

### S1PR1 siRNA transfection

siRNAs and Lipofectamine 2000 were incubated with cells when the cells reached suitable confluence. The S1PR1-siRNAs were purchased from Ribo-Bio (Guangzhou, China). Here were the sequences of the siRNAs:

siRNA for negative control (siNC), ACGUGACACGUUCGGAGAATT; siRNA1 for silencing S1PR1 (si1), CGCCTCTTCCTGCTAATCA; and siRNA2 for silencing S1PR1 (si2), CGGTCTCTGACTACGTCAA.

### MTT assay

Five thousand cells were seeded in 96-well plates per well overnight, the cells were treated with drugs for 72 h. Cells was added with 3-(4,5-Dimethylthiazol-2-*yl*)-2,5-diphenyltetrazolium bromide (MTT) (Sigma, USA) solution and incubated for 4 h at 37 °C. Then, discarded and dissolved with dimethyl sulfoxide (DMSO) (Sigma, USA). Finally, measured the optical densities (ODs) with a microplate reader (Thermo MultisKan FC, USA) at a wavelength of 492 nm.

### Apoptosis assay

5 × 10^5^ cells were seeded in 6-well plates per well overnight and treated with indicated drugs for 48 h. The collected total cells were stained with the Annexin V-PI apoptosis detection kit (Vazyme, China) and analyzed by flow cytometry (Becton Dickinson, USA). FlowJo 6.0 was used to analyze the results. Each assay was performed for three times.

### Calcium-AM/PI dye assays

5 × 10^5^ cells were seeded in 6-well plates per well overnight and treated with the drugs for 72 h. Cells were dyed with PI (Beyotime, China) and calcium-AM (Beyotime, China) for 30 min. Then, captured the images with fluorescence microscope (Olympus Corporation, Japan).

### Real time PCR

Total RNA of the treated cells was extracted by using the RNA Extraction kit (Takara, Japan). Then, the Reverse Transcription System kit (Takara, Japan) was applied to generate the cDNA. Followed by performing the RT-PCR via using the SYBR Green Mix (Takara, Japan). Here were the sequences of the primers:

S1PR1, 5′- GCCTCTTCCTGCTAATCAGCG-3′ (forward), and 5′- GCAGTACAGAATGACGATGGAG-3′ (reverse); and GAPDH, 5′- ATCAATGGAAATCCCATCACCA-3′ (forward) and 5′- GACTCCACGACGTACTCAGCG-3′ (reverse) Each experiment was performed in triplicate.

### Western blotting

5 × 10^5^ cells were seeded in 6-well plates per well overnight, and treated with the drugs for 48 h. Then, cells were added with RIPA lysis buffer contained with the phosphatase inhibitor (Beyotime, China) and protease inhibitor (Beyotime, China), followed by being scraped and collected from the plate. 12% SDS–polyacrylamide gels (Beyotime, China) were applied to separate proteins, then the proteins were transferred to PVDF membranes (Beyotime, China). The PVDF membranes were blocked with 10% (*w*/*v*) nonfat milk for 1 h and incubated with the primary antibodies at 4 °C overnight, then incubated with indicated secondary antibodies. The polyclonal antibodies originated from mouse against S1PR1, P-STAT3, VEGFA, and GAPDH were purchased (Santa Cruz Biotechnology, USA). The polyclonal antibody originated from rabbit against Bax was purchased (Cell Signaling Technology, USA). The secondary antibody used was horseradish peroxidase-conjugated mouse (Proteintech, USA) or rabbit (Proteintech, USA) antibody.

### Cellular uptake assay

3 × 10^5^ cells were seeded onto coverslips in 6-well plates per well overnight, and incubated with free RB (Beyotime, China) or RB@CaP-NPs or RB@cRGD-CaP-NPs for 1 h and 4 h, respectively. Then, cells were fixed and washed, and stained with DAPI (Beyotime, China) for 10 min. The images were captured by using an inverted confocal microscope (Zeiss LSM510, Germany).

### Lysosomal escape assay

The green LysoTracker probe (Beyotime, China) was used to indicate the lysosome, the red RB (Beyotime, China) was used to indicate the drug and the Hoechst 33342 (Beyotime, China) was used to indicate the nucleus. The images of cells were captured by using an inverted confocal microscope (Zeiss LSM510, Germany).

### Immune escape assay

RAW246.7 cells (3 × 10^5^) were seeded onto coverslips in 6-well plates per well overnight, and incubated with free RB (Beyotime, China) or RB@CaP-NPs or RB@cRGD-CaP-NPs for 1 h and 4 h, respectively. Then, cells were fixed and washed, and stained with DAPI (Beyotime, China) for 10 min. The images were captured by using an inverted confocal microscope (Zeiss LSM510, Germany).

### Vascular network formation

HUVECs (2 × 10^4^) were suspended in DMEM and seeded on a 96-well plate precoated with Matrigel (Corning, America). Cells were incubated for 20 h, stained with calcein-AM (Beyotime, China) for 10 min and imaged to determine network formation using a fluorescence microscope at 5× magnification. Then, calculated the polygonal area formed by the endothelial cell network in each view, and analyzed the final number using GraphPad Prism 8.0 software.

### In vitro wounding healing assay

To test the invasive behavior of treated cells, 6 × 10^5^ cells were plated in 6-well plates per well overnight to obtain a confluent monolayer. The monolayer was scratched in a straight line via using a 10 µl pipette tip to create a “wound”. The debris was removed by washing with PBS twice, then added with 2 ml fresh DMEM medium containing the drugs. The images of Cells were captured under a microscope (Olympus Corporation, Japan) at the indicated days posttreatment. Each experiment was performed in triplicate.

### Long circulation characteristics of NPs in blood

BAF312 was replaced with the RB in the NPs to detect the blood long circulation characteristics of NPs in female SD rats due to the strong fluorescence emission of RB. Three-week-old female SD rats were obtained from the Animal Technology Co., Ltd. (Beijing, China). The rats were administered 200 µl of RB or RB@cRGD-CaP-NPs intravenously. After injection, the rat whole blood was collected at the time points of 0.5 h, 1 h, 2 h, 4 h, 6 h, 24 h, 30 h, 48 h, 54 h, and 72 h, then centrifuged, and added 200 µl to 96-well black plate (Corning, USA). A microplate reader (SpectraMax Gemini EM, USA) was used to obtain the optical densities (ODs).

### The tumor targeting abilities of NPs in vivo

BAF312 was replaced with the lipophilic tracer Dir in the NPs to detect the biodistribution of NPs in MDA-MB-231 tumor-bearing female BALB/c nude mice due to the strong fluorescence emission of Dir in the NIR region (*λex*/*λem*: 748/780 nm). 2 × 10^6^ cells were subcutaneously injected into the upper right backs of 4 weeks old mice. Once the tumors volume reached 200 mm^3^, the mice were administered 100 μl of Dir@CaP-NPs or Dir@cRGD-CaP-NPs at a Dir equivalent dose of 0.5 μg/ml intravenously. After injection, the in vivo imaging system (Berthold Technologies, Germany) was used to obtain the whole-body fluorescence images at the time points of 2 h, 12 h, 24 h, and 96 h. The mice were sacrificed and dissected 96 h after the injection to examine the NP distribution in the major organs (spleen, heart, liver, lung, and kidney).

### Anticancer activity in vivo

Three-week-old BALB/c female mice were obtained from the Animal Technology Co., Ltd. (Beijing, China). MDA-MB-231 tumor-bearing mouse models were constructed by subcutaneously injecting 2 × 10^6^ cells into the right mammary fat pad of mice. All animal experiments complied with the relevant ethical regulations for animal testing and research and were approved by the Institutional Animal Care and Use Committee at Renji Hospital, School of Medicine, Shanghai Jiao Tong University (Shanghai, China). When the tumor volume reached 200 mm^3^, the mice were administered with PBS, BAF312 (5 mg kg^−1^), BAF312 at a high dose (10 mg kg^−1^), BAF312@CaP-NPs and BAF312@cRGD-CaP-NPs at a BAF312-equivalent dosage of 5 mg kg^−1^ three times per week (n = 6 per group) intravenously, and the tumor sizes and body weights of the mice were also measured and recorded. On the 28th day, mice were sacrificed, and the tumors and major organs (heart, liver, spleen, lung and kidney) were obtained. The cleaned tumors were weighed and fixed with 4% paraformaldehyde and then sectioned. Immunohistochemistry (IHC) was performed using standard methods. The sections were stained with TUNEL, CD31, Ki67, S1PR1, P-STAT3, VEGFA, and Bax antibodies. Among them, the TUNEL, CD31, and Ki67 antibodies were purchased from Beyotime (China). All of the major organs were also fixed, sectioned and examined by H&E staining.

### Micro-computed tomography (micro-CT)

MDA-MB-231 tumor-bearing female mice of which treated with certain drugs were sacrificed, the limbs of mice were obtained and fixed with 90% ethanol for 24 h. The CT images of limbs were scanned by using a micro-CT imaging system (Belgium).

### TCGA database analysis

We downloaded the S1PR1 expression data and the related information of patients with breast cancer from the TCGA database (https://cancergenome.nih.gov/) and analyzed them. The related information of patients with breast cancer included survival times and TNM stage of breast cancer. The selected patients with breast cancer were assigned into two groups according to the medium value of S1PR1 mRNA expression. The survival duration curve of patients with breast cancer related to the S1PR1 mRNA expression were analyzed via using GraphPad Prism 8.0 software.

### Oncomine database analysis

S1PR1 and VEGFA mRNA expression data of cancer and normal tissues of patients with breast cancer were downloaded from the Oncomine database (https://www.oncomine.org/resource/main.html). mRNA expression in cancer and normal tissues of patients with breast cancer were analyzed via using GraphPad Prism 8.0 software.

### Cancer cell line encyclopedia (CCLE) database analysis

The mRNA expression data of MCF-7 and MDA-MB-231 cells were downloaded directly from the Cancer Cell Line Encyclopedia (http://www.betastasis.com/tissues/cancer_cell_line_encyclopedia/). The mRNA expression of S1PR1, STAT3 and VEGFA in two cell lines was analyzed using GraphPad Prism 8.0 software.

### String database analysis

The relationships among the S1PR1, STAT3 and VEGFA proteins were analyzed by the STRING database and showed that S1PR1, STAT3, and VEGFA were connected with each other, with a PPI confidence value of 0.013.

### Statistical analysis

GraphPad Prism 8.0 software were used to analyze the data, and P values < 0.05 were considered statistically significant.

## Supplementary Information

**Additional file 1: Figure S1.** CCLE database shows that the protein expression levels of S1PR1, STAT3, and VEGFA in MDA-MB-231 cells are much higher than those in MCF-7 cells. **Figure S2.** MTT assay shows that MCF-7 cells are more sensitive to doxorubicin and cisplatin than MDA-MB-231 cells. **Figure S3**. MTT assay shows that the proliferation of MCF-7-siRNAs and MDA-MB-231-siRNAs was downregulated compared with MCF-7-siNC and MDA-MB-231-siNC, respectively. **Figure S4.** MTT assay shows that the proliferation of HUVEC-siRNAs was downregulated compared with that of HUVEC-siNC. **Figure S5.** NMR spectra of DSPE-PEG-cRGD and DSPE-PEG2000-COOH. **Figure S6.** cRGD-modified nanoparticle can exist in the blood circulation for a longer time. Changes of SD rat serum fluorescence value with time after injecting the RB or RB@cRGD-CaP-NPs into the tail vein. Mean ± SEM, n = 3, **P < 0.01, ***P < 0.001. **Figure S7.** BAF312@cRGD-CaP-NPs escape from the immune cells. Blue fluorescence indicates the nucleus; Red fluorescence indicates the RB or RB@CaP-NPs or RB@cRGD-CaP-NPs. **Figure S8.** BAF312@cRGD-CaP-NPs inhibit the migration of HUVECs. (a) Wound healing assay analyzes the migration of HUVECs for 3 days. (b) The statistical results of the wound healing rate for HUVECs. Mean ± SEM, n = 3, **P < 0.01, ***P < 0.001. **Figure S9.** BAF312@cRGD-CaP-NPs inhibit the migration of MCF-7 and MDA-MB-231 cells. (a) Wound healing assay analyzes the migration of MCF-7 cells for 3 days. (b) Wound healing assay was used to analyze the migration of MDA-MB-231 cells after 2 days. (c) The statistical results of the wound healing rate for MCF-7 cells. (d) The statistical results of the wound healing rate for MDA-MB-231 cells. **Figure S10.** Hematoxylin–eosin (H&E) staining shows that the major organs (heart, liver, spleen, lung, and kidney) were not damaged. **Figure S11.** Micro-CT indicates that nanoparticles can protect elderly female nude mice from fractures. The red arrow shows the area of fractures.

## Data Availability

All data about this study are included in this published article and its additional file.

## Abbreviations

BAF312: Siponimod
S1PR1: Sphingosine 1 phosphate receptor 1
VEGFA: Vascular endothelial growth factor A
P-STAT3: Phosphorylated signal transducer and activator of transcription 3
BRCA: Breast cancer
TNBC: Triple-negative breast cancer
MTT: 3-(4,5-Dimethylthiazol-2-yl)-2,5-diphenyltetrazolium bromide
CaP: Calcium and phosphate
DSPE-PEG2000-COOH: 1,2-Distearoyl-sn-glycero-3-phosphoethanolamine-N-[carboxy(polyethylene glycol)-2000]
DSPE-PEG2000-cRGD: 1,2-Dipalmitoyl-sn-glycero-3-phosphoethanolamine-N-(polyethylene glycol)-c(RGDyk)
NPs: Nanoparticles
TCGA: The Cancer Genome Atlas
PBS: Phosphate-buffered saline
