# Supplementary material for: The targetable nanoparticle BAF312@cRGD-CaP-NP represses tumor growth and angiogenesis by downregulating the S1PR1/P-STAT3/VEGFA axis in triple-negative breast cancer
Source: J Nanobiotechnology. 2021 May 31;19:165. doi: 10.1186/s12951-021-00904-6 (PMC8167992; doi:10.1186/s12951-021-00904-6)
Supplement: Supplementary file 1 — Additional file 1: Figure S1. CCLE database shows that the protein expression levels of S1PR1, STAT3, and VEGFA in MDA-MB-231 cells are much higher than those in MCF-7 cells. Figure S2. MTT assay shows that MCF-7 cells are more sensitive to doxorubicin and cisplatin than MDA-MB-231 cells. Figure S3. MTT assay shows that the proliferation of MCF-7-siRNAs and MDA-MB-231-siRNAs was downregulated compared with MCF-7-siNC and MDA-MB-231-siNC, respectively. Figure S4. MTT assay shows that the proliferation of HUVEC-siRNAs was downregulated compared with that of HUVEC-siNC. Figure S5. NMR spectra of DSPE-PEG-cRGD and DSPE-PEG2000-COOH. Figure S6. cRGD-modified nanoparticle can exist in the blood circulation for a longer time. Changes of SD rat serum fluorescence value with time after injecting the RB or RB@cRGD-CaP-NPs into the tail vein. Mean ± SEM, n = 3, **P < 0.01, ***P < 0.001. Figure S7. BAF312@cRGD-CaP-NPs escape from the immune cells. Blue fluorescence indicates the nucleus; Red fluorescence indicates the RB or RB@CaP-NPs or RB@cRGD-CaP-NPs. Figure S8. BAF312@cRGD-CaP-NPs inhibit the migration of HUVECs. (a) Wound healing assay analyzes the migration of HUVECs for 3 days. (b) The statistical results of the wound healing rate for HUVECs. Mean ± SEM, n = 3, **P < 0.01, ***P < 0.001. Figure S9. BAF312@cRGD-CaP-NPs inhibit the migration of MCF-7 and MDA-MB-231 cells. (a) Wound healing assay analyzes the migration of MCF-7 cells for 3 days. (b) Wound healing assay was used to analyze the migration of MDA-MB-231 cells after 2 days. (c) The statistical results of the wound healing rate for MCF-7 cells. (d) The statistical results of the wound healing rate for MDA-MB-231 cells. Figure S10. Hematoxylin–eosin (H&E) staining shows that the major organs (heart, liver, spleen, lung, and kidney) were not damaged. Figure S11. Micro-CT indicates that nanoparticles can protect elderly female nude mice from fractures. The red arrow shows the area of fractures. [file 12951_2021_904_MOESM1_ESM.docx]

Supporting Information

The targetable nanoparticle BAF312@cRGD-CaP-NP represses tumor growth and angiogenesis by downregulating the S1PR1/P-STAT3/VEGFA axis in triple-negative breast cancer

Ke Gong^1^, Juyang Jiao^2^, Chaoqun Xu^3^, Yang Dong^1^, Dongxiao Li^3^, Di He^1^, De Zhao^1^, Jian Yu^1^, Ying Sun^1^, Wei Zhang^1*^, Min Bai^4*^ and Yourong Duan^1*^


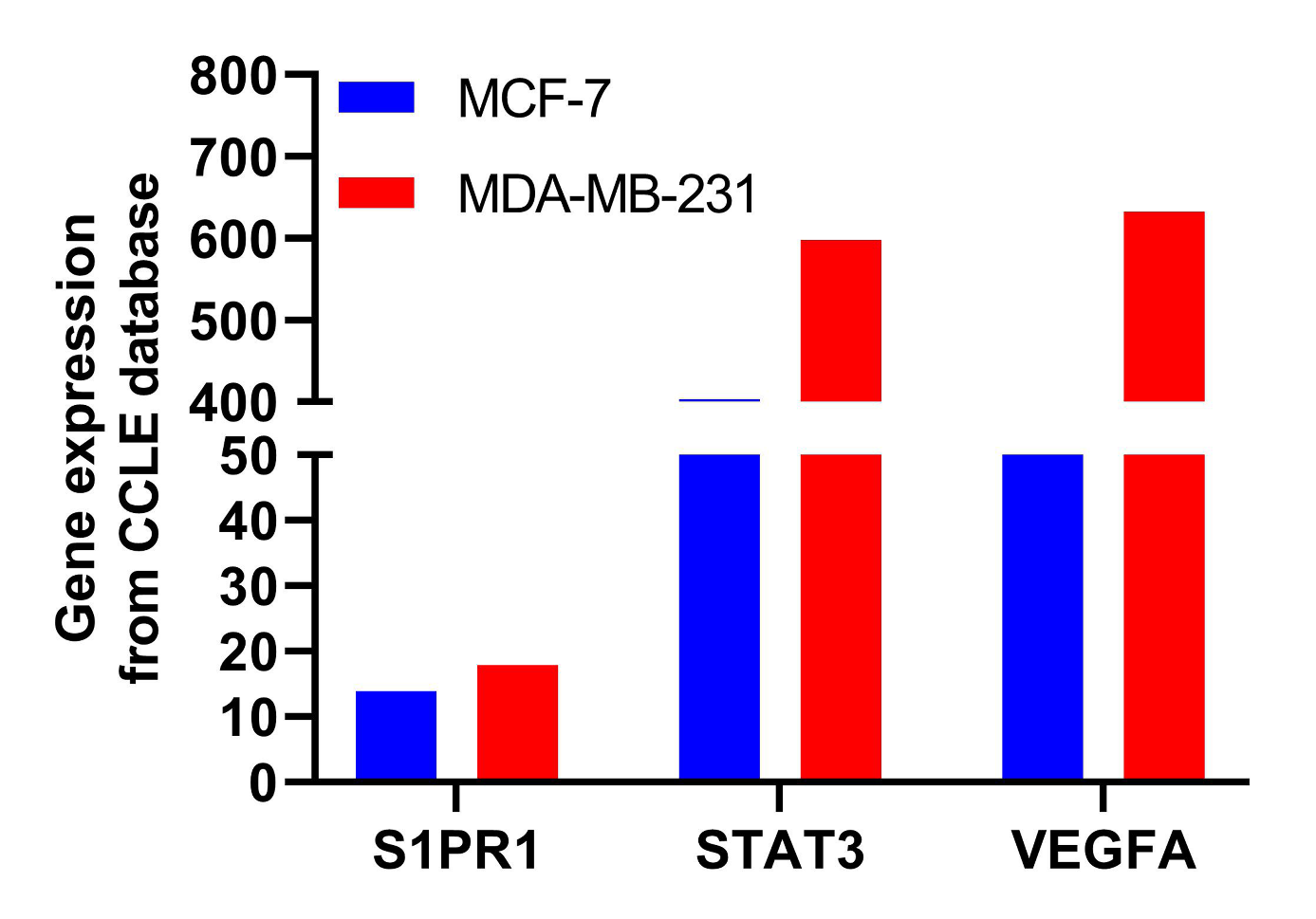


**Figure S1** CCLE database shows that the protein expression levels of S1PR1, STAT3, and VEGFA in MDA-MB-231 cells are much higher than those in MCF-7 cells.


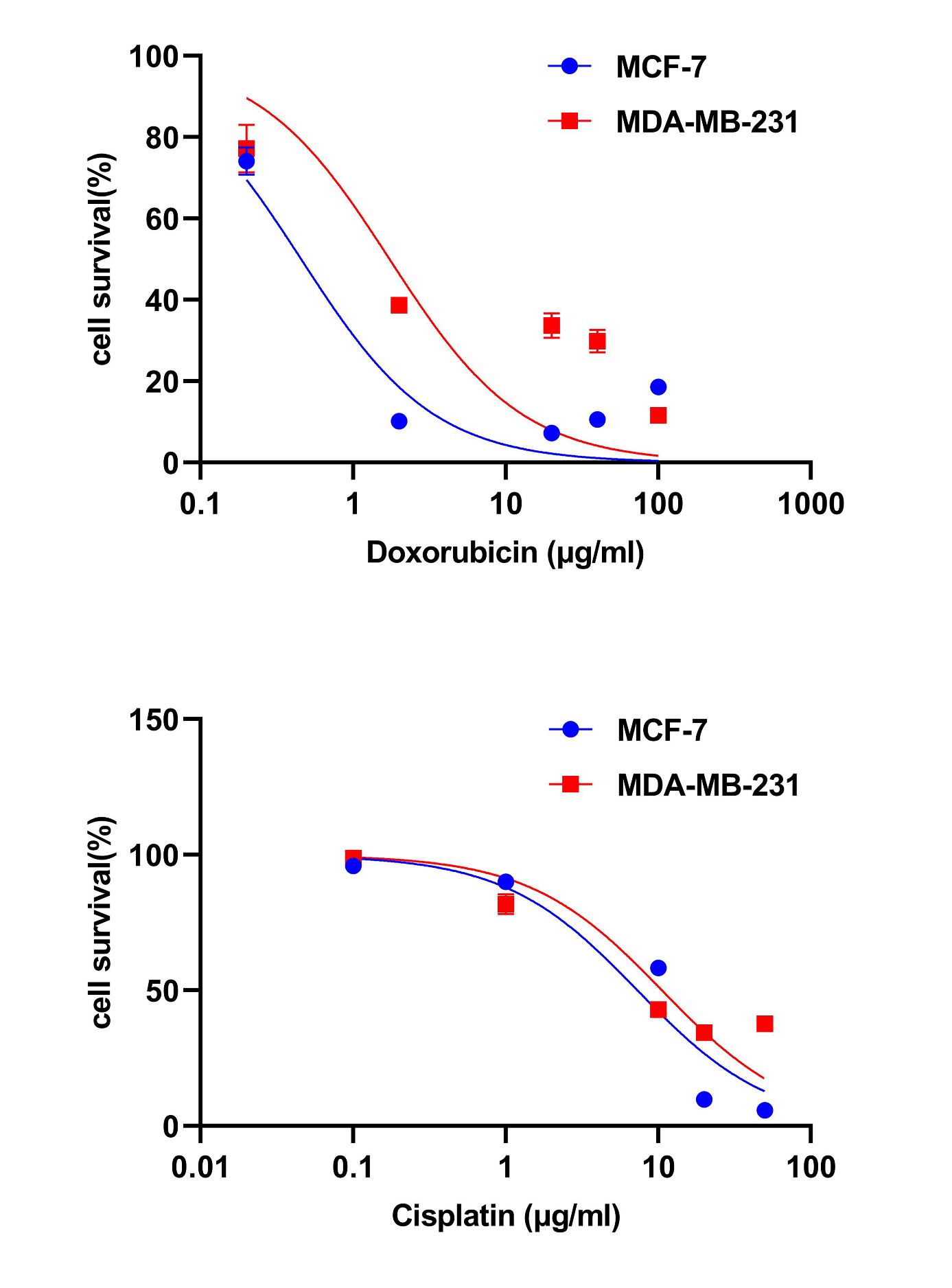


**Figure S2** MTT assay shows that MCF-7 cells are more sensitive to doxorubicin and cisplatin than MDA-MB-231 cells.


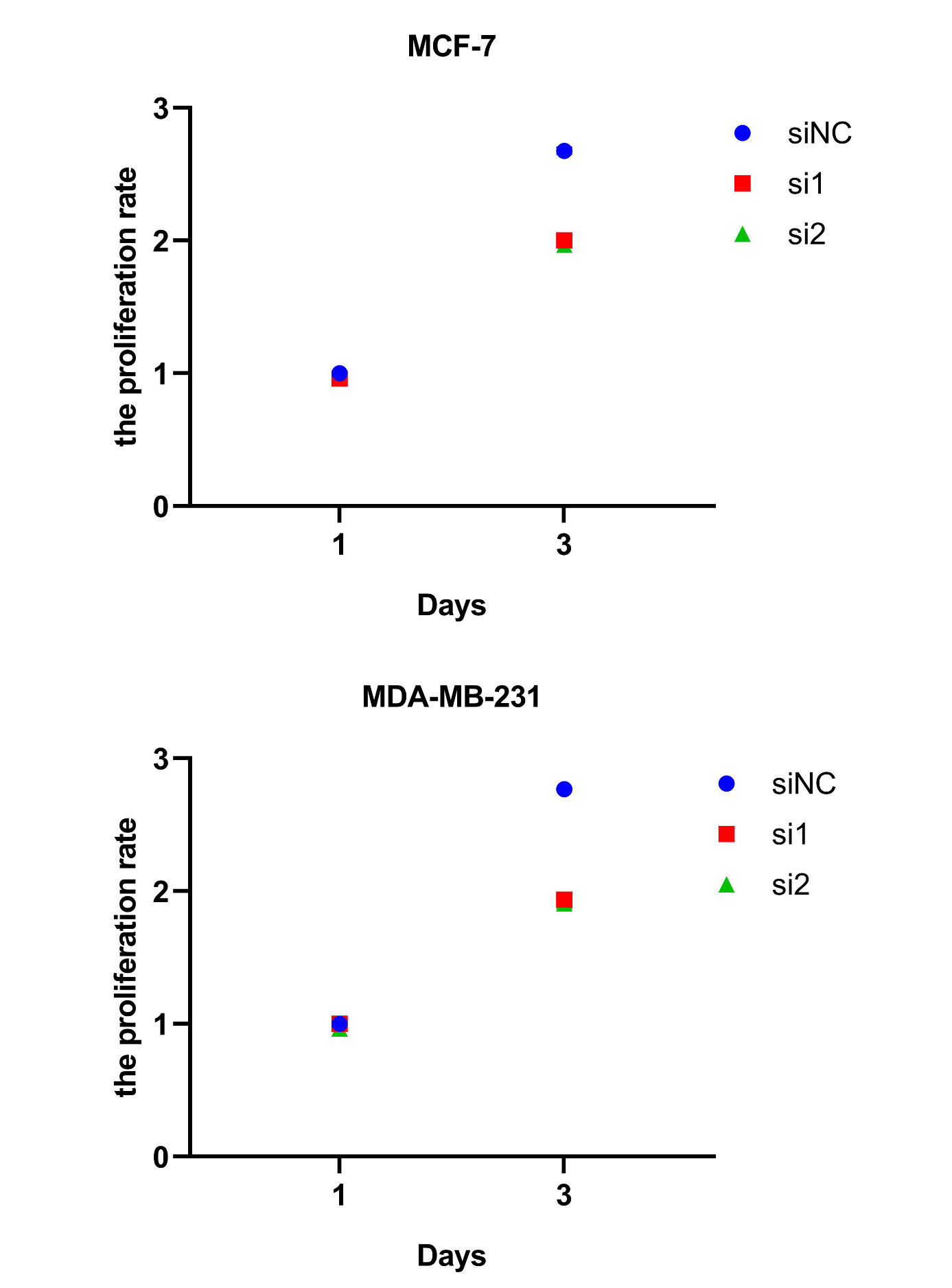


**Figure S3** MTT assay shows that the proliferation of MCF-7-siRNAs and MDA-MB-231-siRNAs was downregulated compared with MCF-7-siNC and MDA-MB-231-siNC, respectively.


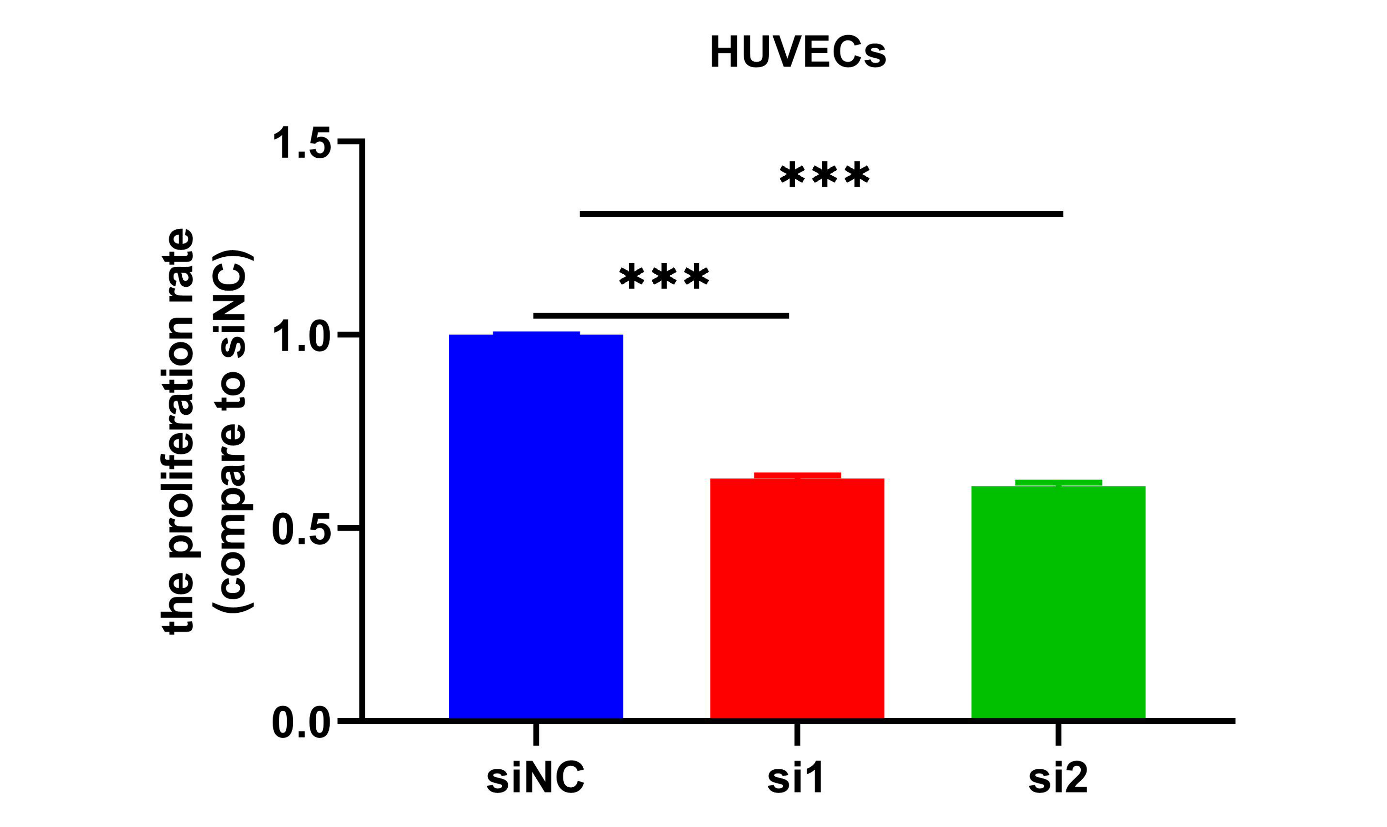


**Figure S4** MTT assay shows that the proliferation of HUVEC-siRNAs was downregulated compared with that of HUVEC-siNC. Mean ± SEM, n = 6, *** P < 0.001.


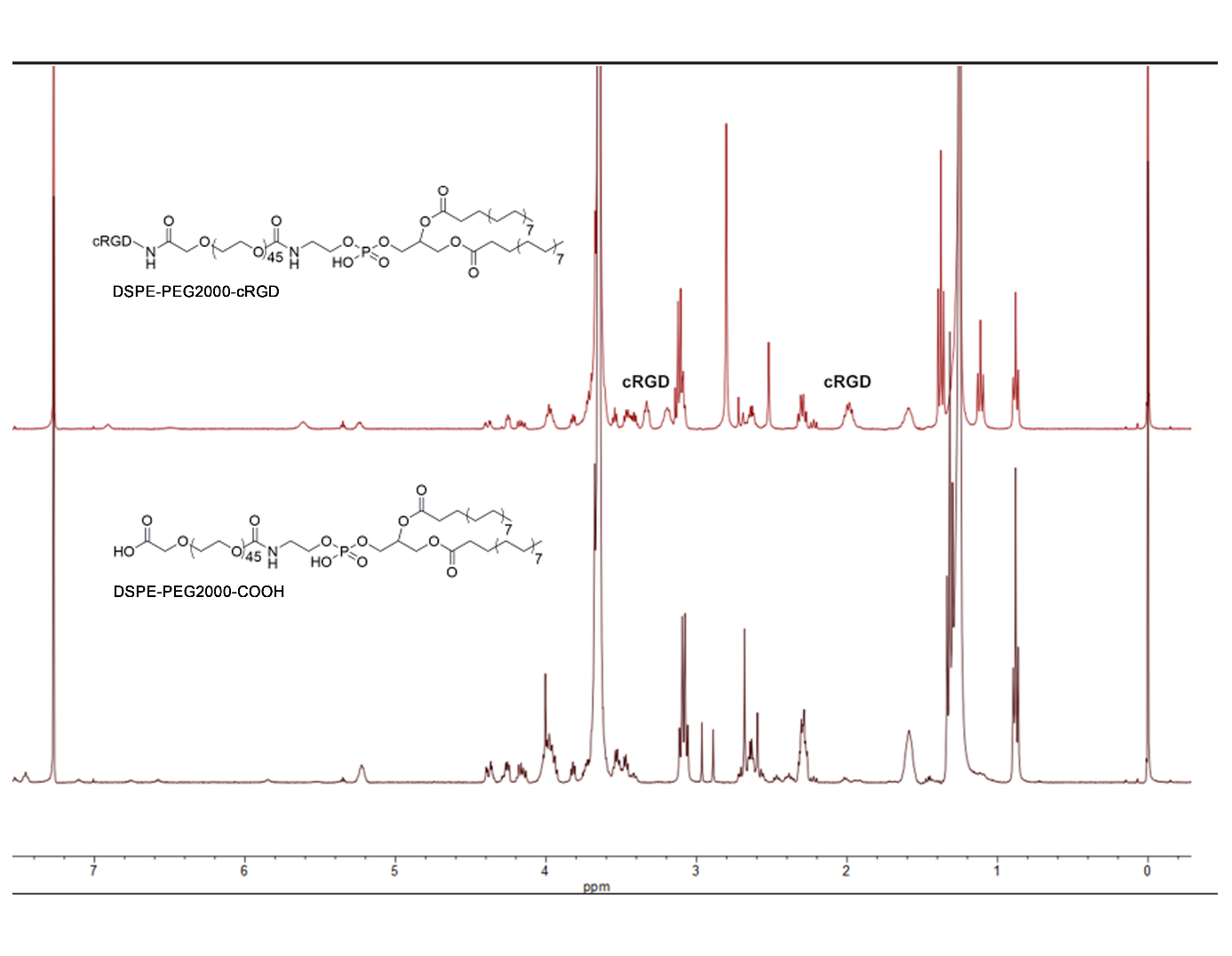


**Figure S5** NMR spectra of DSPE-PEG-cRGD and DSPE-PEG2000-COOH.


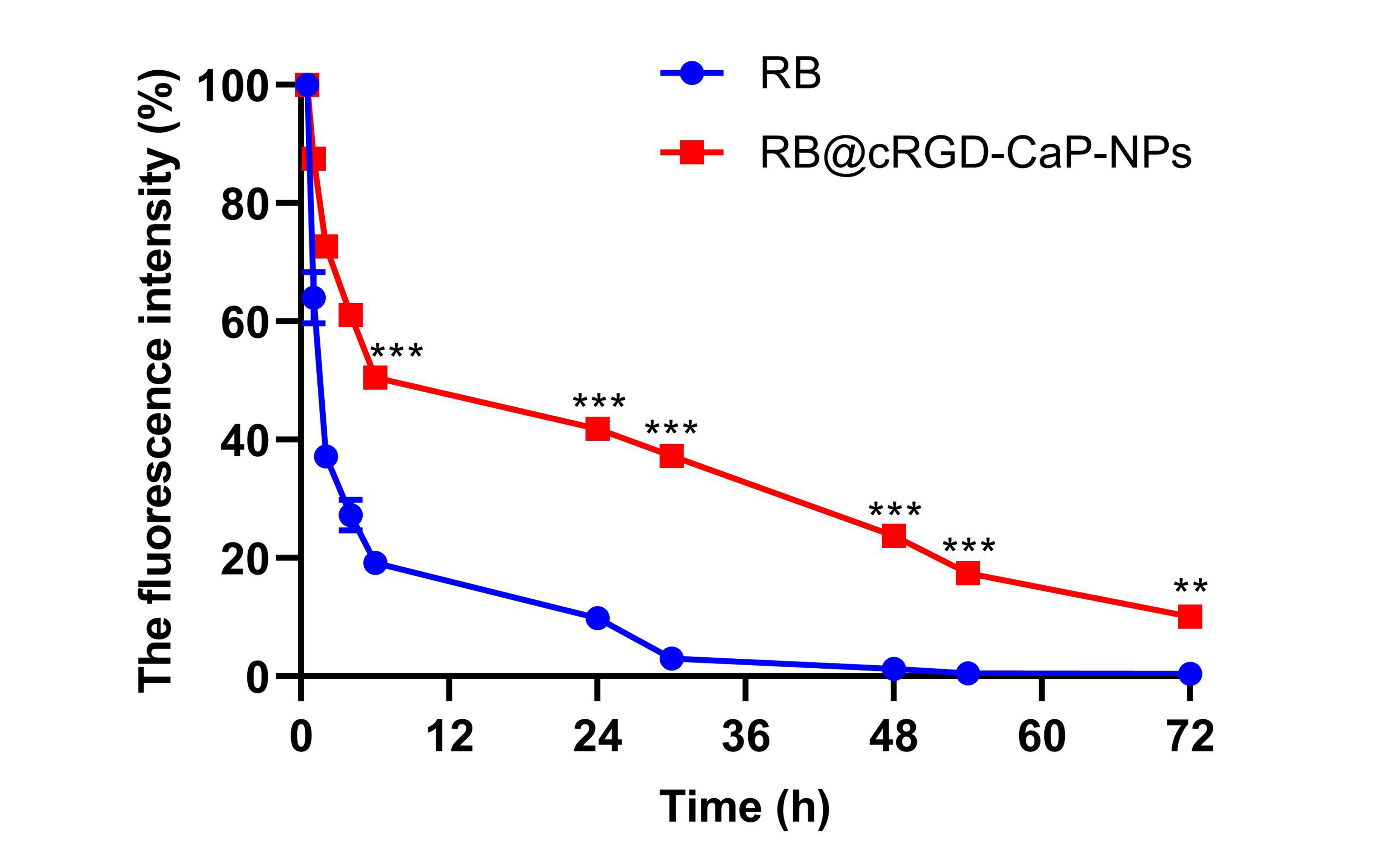


**Figure S6** cRGD-modified nanoparticle can exist in the blood circulation for a longer time. Changes of SD rat serum fluorescence value with time after injecting the RB or RB@cRGD-CaP-NPs into the tail vein. Mean ± SEM, n = 3, ** P < 0.01, *** P < 0.001.


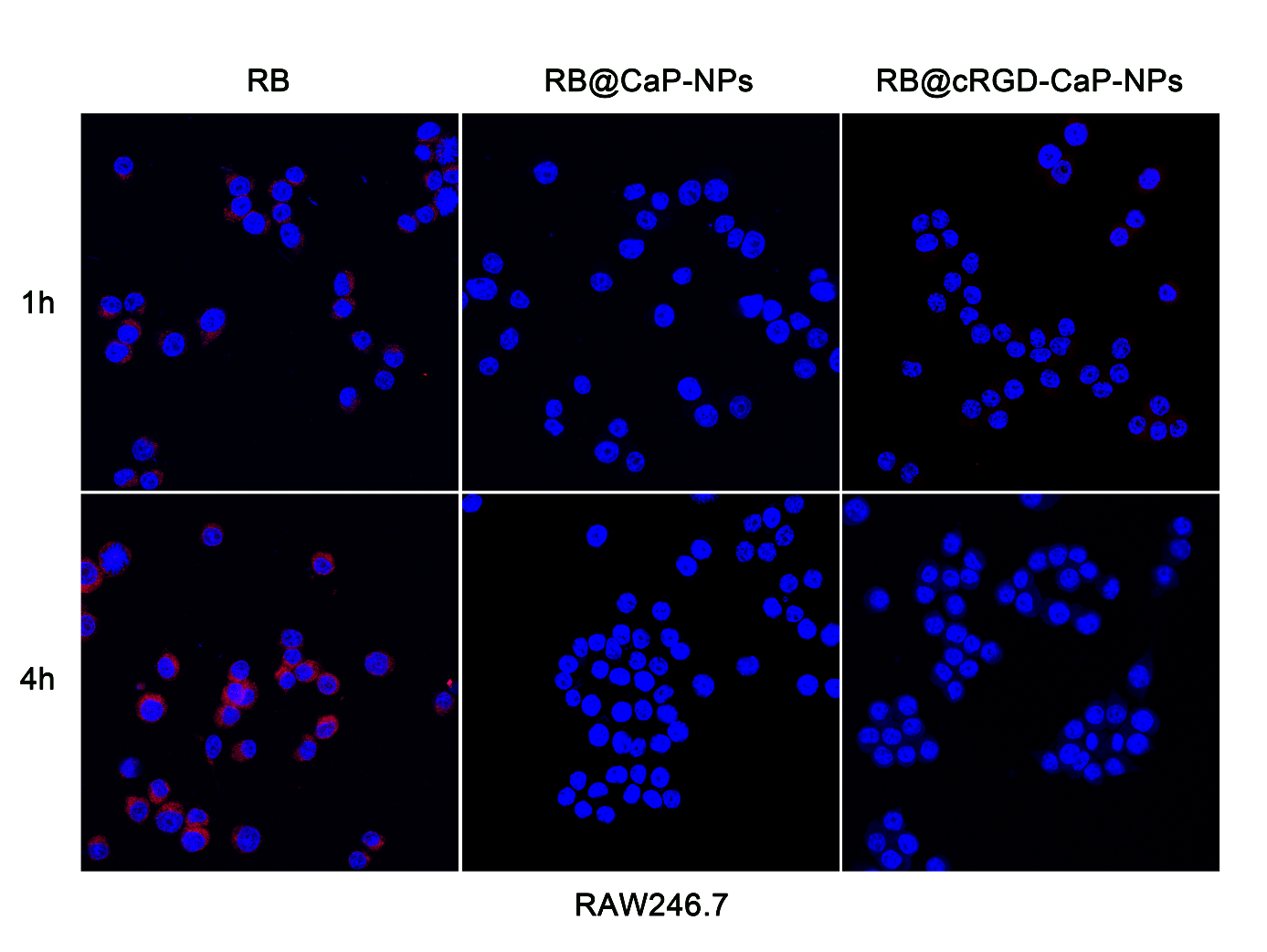


**Figure S7** BAF312@cRGD-CaP-NPs escape from the immune cells. Blue fluorescence indicates the nucleus; Red fluorescence indicates the RB or RB@CaP-NPs or RB@cRGD-CaP-NPs.


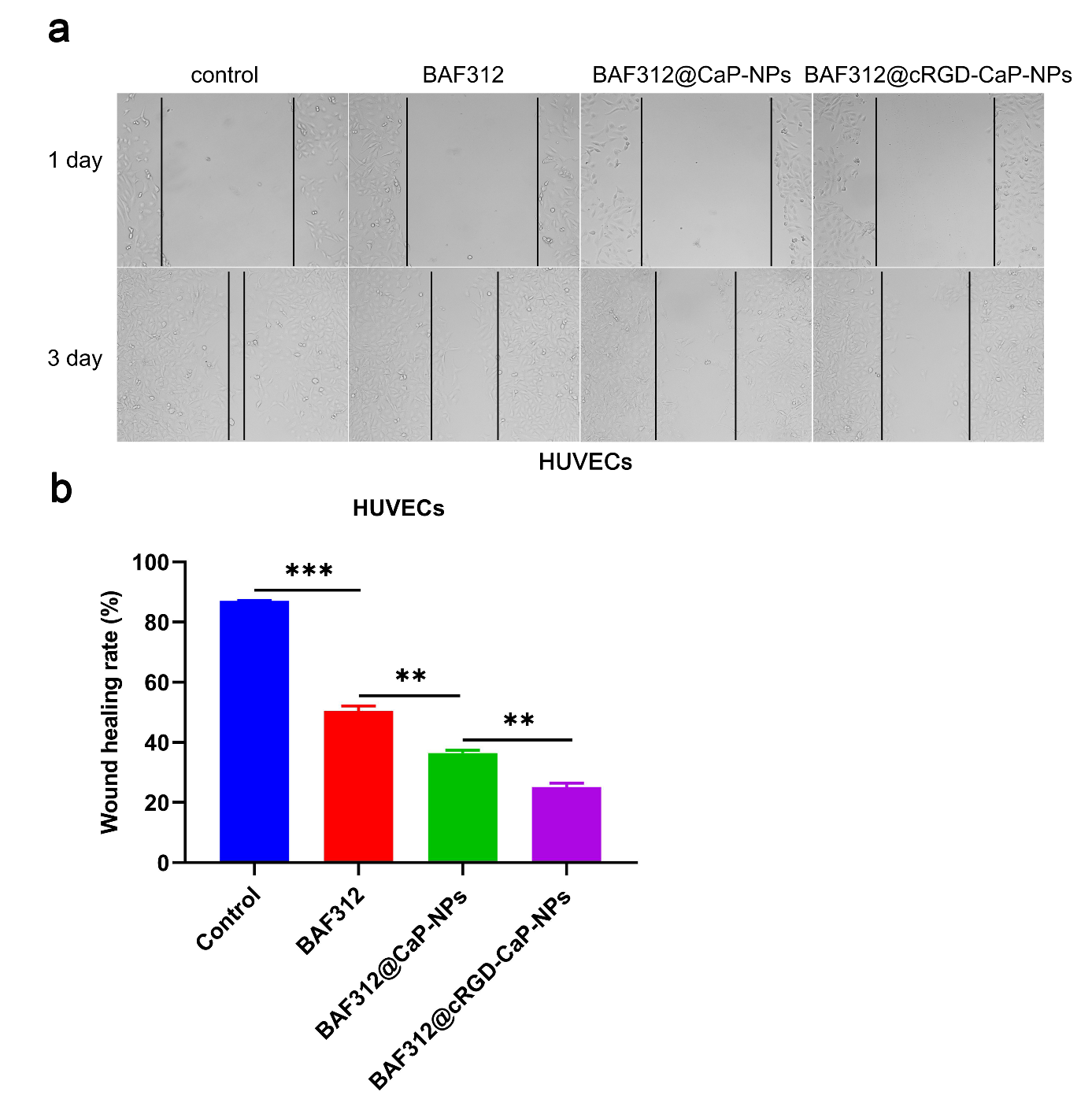


**Figure S8** BAF312@cRGD-CaP-NPs inhibit the migration of HUVECs. (**a**) Wound healing assay analyzes the migration of HUVECs for 3 days. (**b**) The statistical results of the wound healing rate for HUVECs. Mean ± SEM, n = 3, ** P < 0.01, *** P < 0.001.


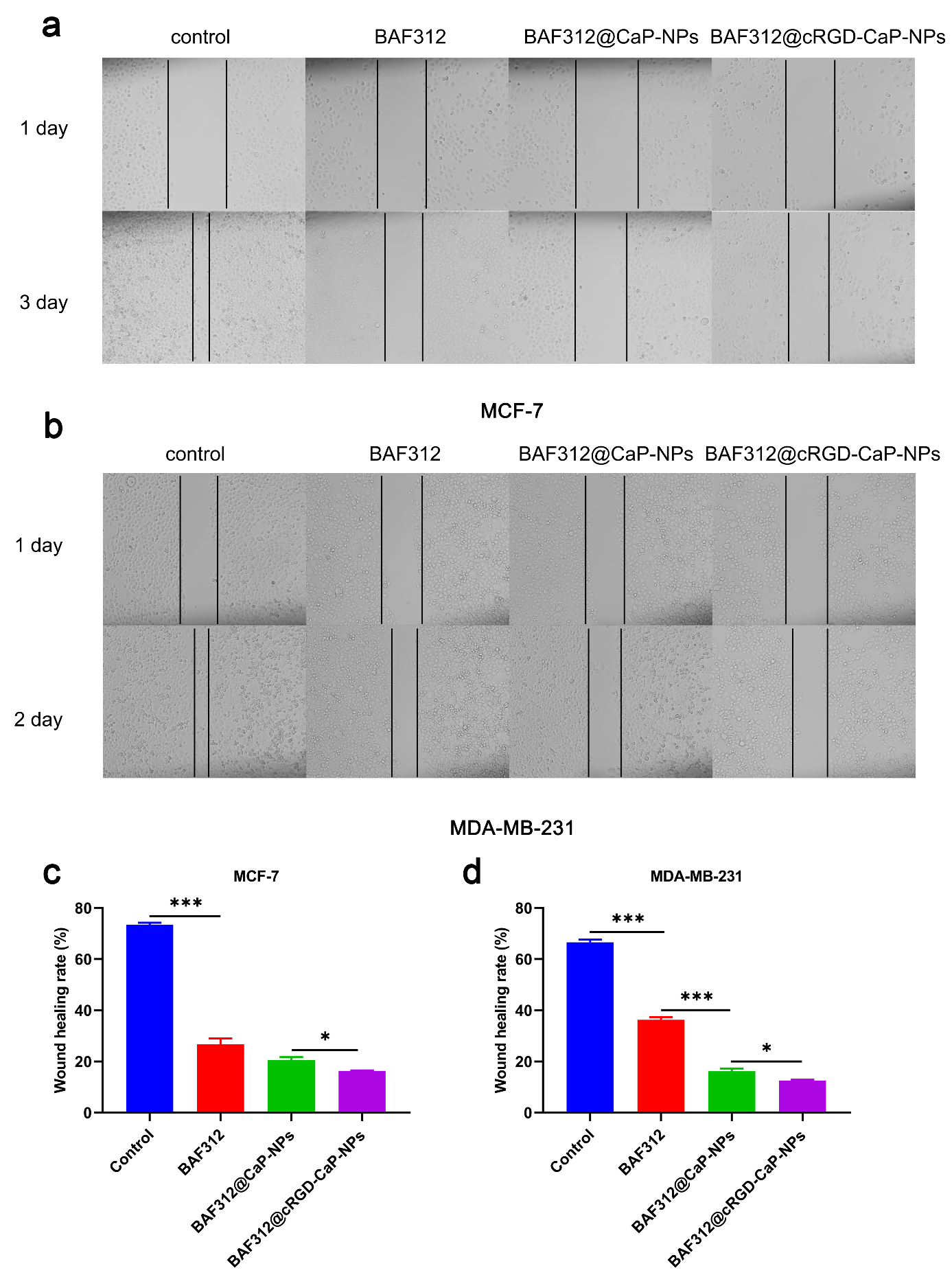


**Figure S9** BAF312@cRGD-CaP-NPs inhibit the migration of MCF-7 and MDA-MB-231 cells. (**a**) Wound healing assay analyzes the migration of MCF-7 cells for 3 days. (**b**) Wound healing assay was used to analyze the migration of MDA-MB-231 cells after 2 days. (**c**) The statistical results of the wound healing rate for MCF-7 cells. Mean ± SEM, n = 3, * P < 0.05, *** P < 0.001. (**d**) The statistical results of the wound healing rate for MDA-MB-231 cells. Mean ± SEM, n = 3, * P < 0.05, *** P < 0.001.


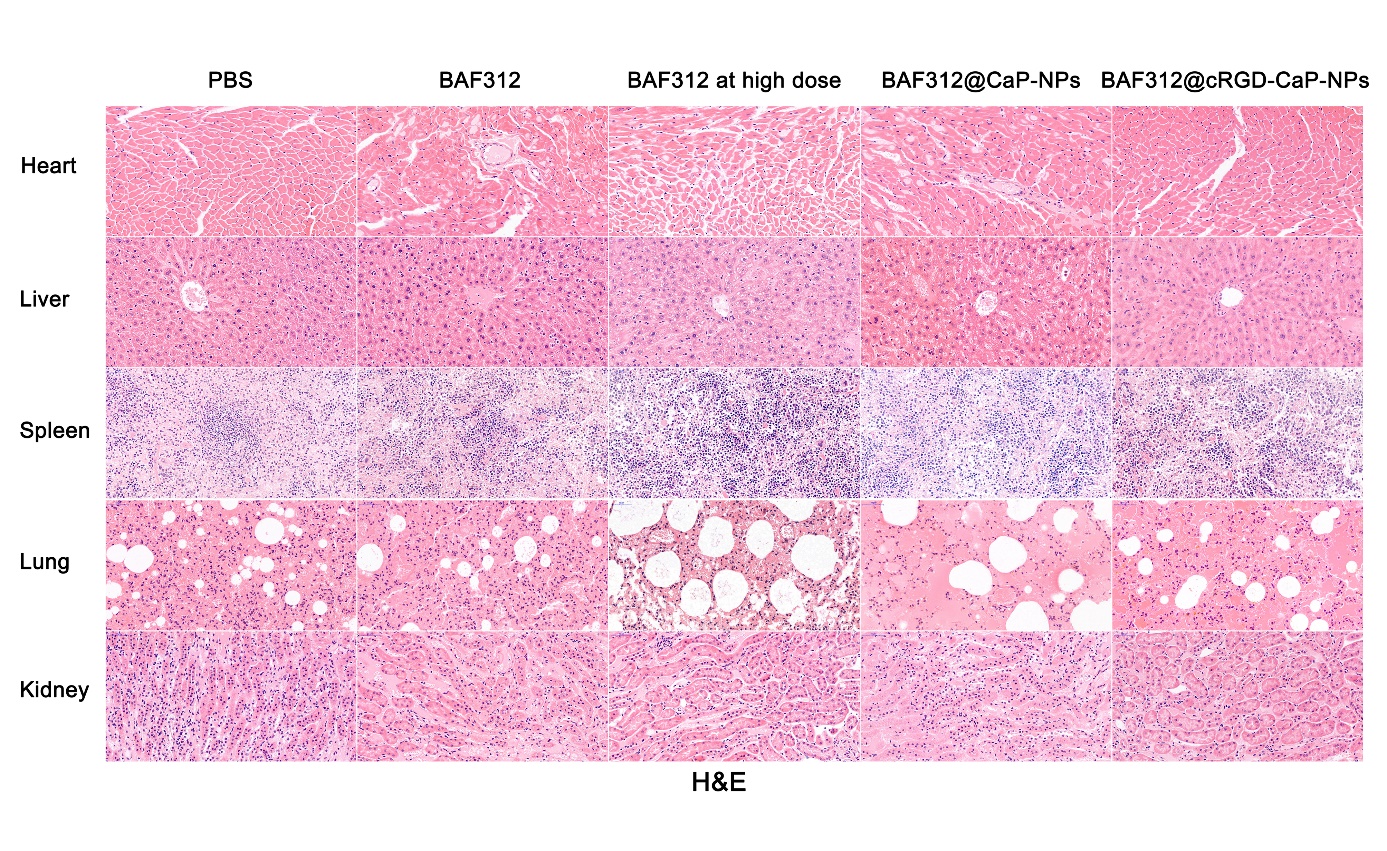


**Figure S10** Hematoxylin-eosin (H&E) staining shows that the major organs (heart, liver, spleen, lung, and kidney) were not damaged.


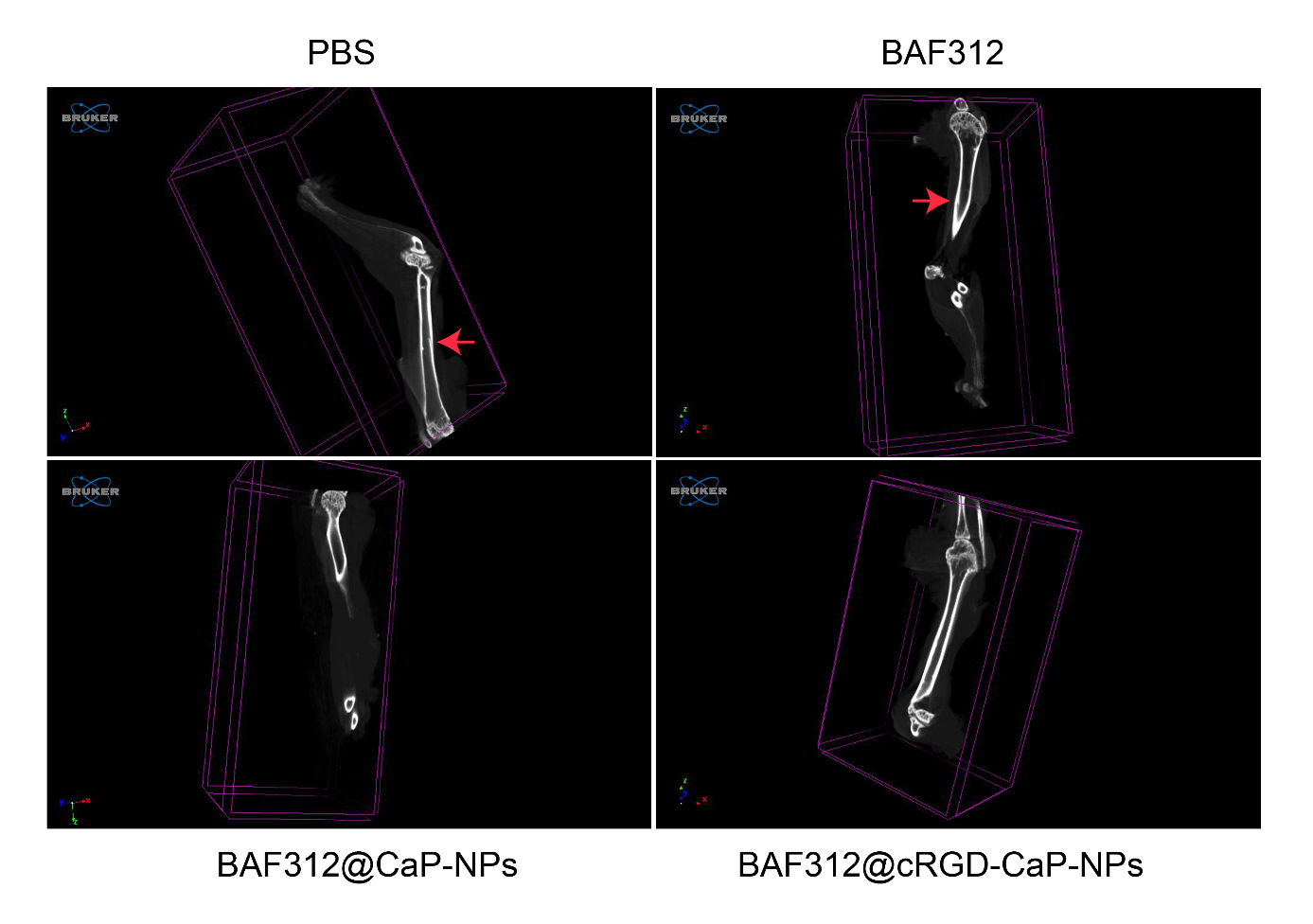


**Figure S11** Micro-CT indicates that nanoparticles can protect elderly female nude mice from fractures. The red arrow shows the area of fractures.
